# Shared immune dysregulation in systemic lupus erythematosus and colorectal cancer: a multi-omics guided discovery of DNASE1L3-centric efferocytosis deficiency

**DOI:** 10.3389/fimmu.2026.1775776

**Published:** 2026-02-26

**Authors:** Hui Guan, Chengzi Tian, Ming Zhong, Lihuan Zhang, Wenjing Wang, Mingcheng Huang, Duo Chen

**Affiliations:** 1 Department of Radiation Oncology, The First Affiliated Hospital of Zhengzhou University, Zhengzhou, Henan, China; 2 Department of Gynecology, The First People’s Hospital of Yunnan Province, The Affiliated Hospital of Kunming University of Science and Technology, Kunming, Yunnan, China; 3 Department of General Surgery, The Seventh Affiliated Hospital of Sun Yat-sen University, Shenzhen, Guangdong, China; 4 Center for Translational Medicine, The First Affiliated Hospital of Zhengzhou University, Zhengzhou, Henan, China; 5 Department of Biochemistry, SUSTech Homeostatic Medicine Institute, School of Medicine, Southern University of Science and Technology, Shenzhen, Guangdong, China; 6 Department of Nephrology, Center of Kidney and Urology, The Seventh Affiliated Hospital of Sun Yat-sen University, Shenzhen, Guangdong, China; 7 Department of Endocrinology and Metabolism, The First Affiliated Hospital of Zhengzhou University, Zhengzhou, Henan, China

**Keywords:** colorectal cancer, DNASE1L3, efferocytosis, macrophages, systemic lupus erythematosus

## Abstract

**Background:**

Although immune dysregulation is implicated in both autoimmune diseases and cancer, comparative pathogenesis and immune response mechanisms between systemic lupus erythematosus (SLE) and colorectal cancer (CRC) remain elusive. This study identifies common molecular biomarkers and pathogenic pathways shared between SLE and CRC via multi-omics analysis.

**Methods:**

Integrated datasets including SLE (GSE61635, GSE50772, GSE142016) and CRC cohorts (GSE39582, GSE17536, E-MTAB-8107, COAD, READ), were analyzed. Differential expression analysis identified shared genes, and machine learning screened four hub prognostic genes. A risk model based on these genes was constructed to evaluate SLE screening and CRC prognosis. Multi-omics approaches explored mutational profiles, immune infiltration, drug sensitivity, and signaling pathways. Reverse transcription-quantitative polymerase chain reaction (RT-qPCR), staining were performed to confirmed the expression levels of the hub genes. The impact of DNASE1L3 knockdown in the human monocyte cell line THP-1 on phagocytosis of apoptotic intestinal epithelial cell line NCM460 was evaluated using RT-qPCR and flow cytometry.

**Results:**

We identified 58 shared differentially expressed genes (DEGs) between SLE and CRC, enriched in apoptosis, oxidative phosphorylation, and immune infiltration. Machine learning highlighted four hub genes (*DNASE1L3*, *PTPN14*, *SELENBP1*, *ECRG4*), forming a robust predictive model for SLE occurrence and CRC prognosis. Shared immune infiltration patterns and small-molecule drug candidates were observed. Single-cell and spatial transcriptomic analyses revealed DNASE1L3 predominantly in myeloid cells. Cellular experiments revealed that reduced DNASE1L3 levels significantly compromised macrophage efferocytosis of apoptotic cells, accompanied by a decrease in M2 macrophage proportion, an increase in M1 macrophage proportion, diminished LOX enzyme activity, and elevated levels of interleukin-1 β and tumor necrosis factor-α.

**Conclusion:**

SLE and CRC exhibit overlapping DEGs, immune profiles, and signaling pathways. The model based on the shared genes—*DNASE1L3*, *PTPN14*, *SELENBP1*, and *ECRG4*—offers novel insights for precise intervention in both diseases.

## Introduction

Systemic lupus erythematosus (SLE) is a chronic, systemic, autoimmune, inflammatory, connective tissue disease (1). The global incidence of SLE varies from about 1.5 to 11 per 100,000 person-years, with a global prevalence of 13 to 7,713.5 per 100,000 people (2). The pathogenesis mechanism of SLE is complex and is closely related to ultraviolet light exposure, high estrogen, genetics, and drugs (3). The clinical manifestations of SLE are highly heterogeneous and can involve almost every organ, including the skin, joints, lungs, kidneys, nervous system and gastrointestinal tract, etc. (4). In addition to renal failure, cardiovascular disease and infections, tumors are also an important cause of death in SLE. In recent years, growing evidence has revealed a strong association between SLE and cancer. Several large cohort studies have observed a significantly increased risk of malignancy in patients with SLE (5, 6).

In 2022, there were 20 million new tumor cases and over 9.7 million deaths worldwide. Colorectal cancer (CRC) ranked third in cancer incidence and second in cancer-related mortality, with 1,926,118 new cases (9.6% of all cases) and 903,859 deaths (9.3% of all cancer deaths) (7). Genetics, poor dietary and lifestyle, and inflammatory bowel disease were high risk factors for the development of CRC. Several studies revealed SLE was an independent risk factor for CRC (8). Relevant evidence suggests that anti-tumor therapy (e.g. capecitabine and fluorouracil) may induce SLE (9, 10).

Several potential mechanisms may explain the association between SLE and CRC. First, imbalance of lactic acid metabolism, persistent and severe chronic inflammation are common risk factors for both SLE and CRC (11, 12). SLE is typically characterized by immune dysfunction, with the release of pro-inflammatory cytokines (e.g., IL-6, TNF-α, IL-17, etc.), which further provides a pro-tumor inflammatory response environment for the development of CRC. For example, IL-6 activates the JAK/STAT3, RAS/MAPK, and PI3K/AKT signaling pathways and exerts anti-apoptotic and tumorigenic effects (13). The presence of a large number of autoantigens in SLE patients activates Toll-like receptors, which promote inflammation and transform pre-cancerous cells into cancerous cells through the MyD88/NF-κB signaling pathway (14). In addition, therapeutic drugs for SLE patients, such as glucocorticoids, immunosuppressive drugs (e.g., methotrexate, cyclophosphamide), and biologics (e.g., TNF-α tumor inhibitors), increase the risk of tumors (15, 16). Furthermore, high levels of autoantigens and autoantibodies, immune dysregulation, genetic susceptibility, and environmental factors may also be pathogenic mechanisms shared by SLE and CRC.

Numerous studies have shown that the link between autoimmune diseases and cancer is dynamic and bidirectional. Disruption of immune tolerance leads to SLE and dysregulation of immune surveillance leads to tumorigenesis. Immunity, although it appears to function differently at the onset of disease, the underlying mechanisms are shared (17). Patients with SLE combined with tumors have a high disease burden and a poor prognosis. There is also a correlation between the outcome of treatment, long-term survival, and the development of autoimmune diseases in tumor patients. Bruton’s tyrosine kinase inhibitors, IL-6 inhibitors, and IL-38 have been shown to be effective in both SLE and CRC (13, 18, 19). Therefore, the common pathogenic mechanisms and therapeutic targets of SLE and CRC deserve in-depth study.

In this study, we first identified common differentially expressed genes (DEGs) from the GSE39582 and GSE61635 datasets. Subsequently, hub genes were screened through univariate Cox regression, least absolute shrinkage and selection operator (LASSO) regression, and support vector machine-recursive feature elimination (SVM-RFE), establishing sample-specific risk scoring. We then explored shared molecular pathways, immune microenvironment features, and therapeutic compounds between both diseases. A hub gene-based nomogram model demonstrated robust predictive performance for diagnosing SLE and CRC, as well as predicting CRC survival outcomes. The CRC risk score was validated using GSE17536, COAD, and READ datasets, followed by comparisons of biological functions, genomic alterations, and stemness characteristics between high-risk and low-risk CRC populations. Additionally, single-cell datasets (EMTAB-8107 and GSE142016) and spatial transcriptomics were leveraged to investigate hub gene expression patterns and spatial distribution. Finally, hub gene expression and functional relevance was validated *in vitro*. This research elucidates shared biomarkers and pathogenic mechanisms between SLE and CRC, facilitating early disease identification and intervention while guiding personalized therapeutic strategies.

## Methods

### Data collection

GSE61635 and GSE50772 dataset of peripheral blood mononuclear cells (PBMC) from SLE patients, were downloaded from the Gene Expression Omnibus (GEO, https://www.ncbi.nlm.nih.gov/geo/) database. The GSE61635 dataset, comprising 99 SLE samples and 30 control samples, was used as the training cohort. The GSE50772 dataset, including 61 SLE samples and 20 control samples, was used as the validation cohort. Two datasets of CRC patients, including GSE39582 and GSE17536, were retrieved from GEO database as well (20, 21). The dataset of colon adenocarcinoma (COAD) and rectum adenocarcinoma (READ) was obtained from The Cancer Genome Atlas (TCGA) database via UCSC Xena website (https://xena.ucsc.edu/) (22). The GSE39582 dataset, as a training set, consisted of 566 tumor samples and 19 control samples. GSE17536, as well as the COAD and READ datasets, were used as external validation cohorts. GSE17536 comprises 177 tumor samples. The COAD dataset includes 448 tumor and 39 control samples, while the READ dataset consists of 158 tumor and 9 control samples. The expression data from GEO database was generated using the GPL570 platform. Single-cell RNA sequencing data from 7 CRC patients in the E-MTAB-8107 dataset were downloaded from the TISCH2 database (http://tisch.compbio.cn) (23). Single-cell data from 3 SLE patients in the GSE142016 dataset were obtained from the GEO database (24).

### DEGs screening and functional analysis

DEGs in GSE39582 and GSE61635 datasets were screened out and visualized in the form of volcano plots using the “limma” and “ggplot2” packages in R software. |Log2 (fold change)| >1 and adjusted P value ≤ 0.05, with multiple testing correction performed using the Benjamini–Hochberg method were considered to indicate statistical significance. Subsequently, the common DEGs in both CRC and SLE datasets was shown in a Venn diagram. The expression patterns of common DEGs were visualized in the form of heatmaps with “pheatmap” package. Moreover, in order to analyze the biological functions and pathways involved in common genes, Gene Ontology (GO) and Kyoto Encyclopedia of Genes and Genomes (KEGG) enrichment analyses were performed using The Database for Annotation, Visualization, and Integrated Discovery (DAVID, https://davidbioinformatics.nih.gov/), with terms/pathways with adjusted P value (Benjamini–Hochberg) < 0.05 were considered significantly enriched Additionally, a protein-protein interaction (PPI) network was constructed using STRING (https://string-db.org/) to evaluate potential functional associations among these shared genes.

### Connectivity map analysis

Relationships between common DEGs and small molecule therapeutics were explored via the cMAP database (https://clue.io) (25). The 31 upregulated DEGs were incorporated into the cMAP platform, resulting in identification of the top 10 compounds exhibiting maximal enrichment scores. Chemical structure of small molecular compounds was illustrated via ChemSpider database (http://www.chemspider.com) and visualized with Chemdraw software (version 22.0.0.22).

### Machine learning selection of characteristic biomarkers

Signature genes for CRC and SLE were identified using three machine learning methods: univariate Cox regression, LASSO, and SVM-RFE algorithm. First, prognostic genes were screened from common DEGs using univariate Cox regression implemented via the “survival” package. Subsequently, the LASSO regression model was applied using the “glmnet” package, where the hyperparameter lambda (λ) minimized regression coefficients toward zero to exclude weakly correlated features, with optimal parameters determined through 5-fold cross-validation (26). Concurrently, SVM-RFE was employed using the “mlbench” and “caret” packages to iteratively eliminate the least important feature vectors (27). Finally, characteristic genes identified by LASSO and SVM-RFE for CRC and SLE were intersected, yielding shared signature genes for downstream analysis.

### Network analysis of characteristic genes

NetworkAnalyst (http://www.networkanalyst.ca) is a comprehensive web-based tool designed to identify proteins of interest, construct networks, and perform network analysis and visualization (28). The characteristic genes for CRC and SLE were uploaded to the NetworkAnalyst website, mapped to the integrative interaction database to construct correlation networks. The interactions of significant genes with transcription factors (TFs) and microRNAs (miRNAs) were analyzed subsequently. Transcription factor targets were derived from the JASPAR TF binding site profile database. Comprehensive experimentally validated miRNA-gene interaction data were collected from TarBase database. Finally, coregulatory networks among genes, TFs, and miRNA were constructed by Cytoscape software (version 3.10.2).

### Immune infiltration evaluation

The 22 types of immune cell composition in patients and controls were estimated with the cell-type identification by estimating relative subsets of RNA transcripts (CIBERSORT) algorithm (29). The proportion and abundance of immune cells in each sample were shown as the stacked histogram with the “ggplot2” package. Subsequently, the differences between the proportions of immune cells in the both groups were shown in a box plot. Next, the association of the infiltrated immune cells was evaluated with the “corrplot”package and presented as a heatmap with the”pheatmap”package. Finally, the correlation between the four characteristic genes and the infiltrating immune cells was analyzed using the Spearman correlation coefficient and visualized as a heatmap.

### Construction of nomogram model in SLE

The nomogram diagnostic model was constructed for four characteristic genes using the “rms”package. The risk score for each participant of SLE and controls was calculated based on the nomogram model. Receiver operating characteristic (ROC) curves via “pROC” package, violin plots via “ggbiplot” package, calibration curves via “calibration curve” package, and decision curve analysis (DCA) via “rmda” package evaluated the ability of the diagnostic model to discriminate between SLE and controls (30). GSE50772 was used as an external validation cohort to evaluate the robustness of the model.

### Construction of prognostic model in CRC

Based on the normalized expression levels (Expi) and corresponding regression coefficients (Coei) of prognostic genes, the risk score of each patient was calculated according to the following formula:


$$\text{Risk score}=\sum_{i=1}^{N}(Expi\times Coei)$$

First, the diagnostic performance of the four-gene-based risk score for CRC was evaluated using ROC analysis. Subsequently, its prognostic capability for predicting overall survival (OS) of CRC was assessed. CRC patients were divided into high-risk and low-risk groups based on the median cut-off value of the risk score. Subsequently, Kaplan-Meier analyses were performed using the “survival”package to compare the survival of patients in the both groups. Distribution in the proportion, survival time, and survival status of high-risk and low-risk groups in CRC patients were explored with the “ggrisk” package.

Furthermore, univariate and multivariate Cox regression analyses were also conducted to evaluate the impact of risk score and other clinical characteristics (including age, chemotherapy, clinical stage, T/N/M classification) on the prognosis of colorectal cancer patients. ROC, calibration, and DCA curves were evaluated the ability of the prognostic model to predict OS of CRC. The COAD and READ datasets served as external validation cohorts to assess the predictive capability of the nomogram.

### Mutational profiles for CRC

Somatic mutations in the 15 most frequently mutated genes were analyzed using the “maftools” package to compare mutational profiles between high-risk and low-risk cohorts. Furthermore, copy number variation (CNV), specifically gene amplifications and deletions, were assessed in COAD and READ cohorts via “tidyverse” package.

### Stemness analysis for CRC

Stemness gene sets were retrieved from StemChecker website (http://stemchecker.sysbiolab.eu/), which was based on the most comprehensive and updated collection of published stemness signatures defined by expression profiles, RNAi screens, literature curation, computationally derived, Nanog, and transcription factor target gene sets. Then, quantitative analysis of stemness enrichment score of each participant was elucidated with the single-sample gene set enrichment analysis (ssGSEA) method. Adjusted P value < 0.05 was used as the significance threshold.

### Immune response analysis for CRC

The differential expression of immune checkpoint and human leukocyte antigen (HLA) were assessed across high-risk and low-risk populations. The immune score, stromal score, ESTMATE score, tumor purity of each sample was calculated with the “ESTMATE” package (31). In addition, tumor immune dysfunction and exclusion (TIDE) score of each sample was evaluated to predict the possibility of immune escape and immune response occurring in the tumor via the website (http://tide.dfci.harvard.edu) (32). Immunophenotypic score (IPS) data were obtained from The Cancer Immunome Atlas (TICA) database (https://tcia.at/home) (33). The responsiveness to anti-PD-1 or anti-CTLA-4 therapy was evaluated by comparing IPS between high-risk and low-risk groups via the “ggpubr” package.

### Functional enrichment analysis in CRC

The DEGs between the high-risk and low-risk groups in the GSE39582 dataset were defined and presented as the volcano plot. Potential biological functions were examined through GO enrichment analysis via Metascape database (https://metascape.org) (34). Enriched pathways were further integrated using the gene set enrichment analysis (GSEA) was performed with the “clusterProfiler” package, applying thresholds of |normalized enrichment score (NES)| > 1, NOM P value < 0.05, and q value < 0.25. Additionally, gene set variation analysis (GSVA) was conducted to identify pathways enriched in the two risk cohorts via the “GSVA” package.

### Single cell RNA sequencing analysis

Single-cell RNA sequencing data from 7 CRC patients in the E-MTAB-8107 dataset were processed using the “Seurat” package. Cells meeting quality control criteria (featuring more than 400 and fewer than 6000 expressed genes, with a mitochondrial gene percentage below 20%) were retained for subsequent analysis. Data normalization was performed using the “NormalizeData” function. Highly variable genes across cells were identified using the “FindVariableFeatures” function. Subsequently, data scaling was conducted via the “ScaleData” function, followed by principal component analysis (PCA). Cell clustering analysis was performed using the “FindNeighbors” and “FindClusters” functions, and the resulting clusters were visualized by applying uniform manifold approximation and projection (UMAP). Cell-cell communication was analyzed using the “CellChat” package.

We aimed to investigate the expression patterns of the four core genes across eight annotated cell types: epithelial, plasma, T, myeloid, mast, natural killer (NK), fibroblast, and endothelial cells. Furthermore, these cell types were categorized into three functional groups—epithelial, stromal (fibroblasts and endothelial cells), and immune cells (plasma, T, myeloid, mast, and NK cells)—to systematically compare and analyze the expression levels of the four hub genes within these broader functional compartments. Based on the positive or negative expression status of the deoxyribonuclease 1 like 3 (*DNASE1L3*) gene, all cells were stratified into a positive group and a negative group. The proportion of each cell subpopulation within these two groups was calculated to elucidate the expression pattern of *DNASE1L3*. Differential expression analysis was further performed between these two groups, and the results were visualized using a volcano plot. KEGG analysis was conducted on the DEGs to identify significantly enriched signaling pathways. Pseudotime analysis was analyzed using the “monocle” package to investigate developmental trajectories of cells (35). A similar analytical workflow was applied to single-cell RNA sequencing data from 3 SLE patients in the GSE142016 dataset.

### Spatial transcriptome analysis of CRC

Spatial transcriptome analysis of CRC was performed via the Sparkle website (https://grswsci.top/) (36). Briefly, inverse convolutional analysis was employed to accurately assess cellular composition on different spatial localizations. Strict quality control measures were implemented on the collected single-cell transcriptome data based on the number of expressed genes, the count of unique molecular identifiers, and the percentage of mitochondrial RNA in each cell. Subsequently, a signature score matrix was constructed by calculating the average expression of the top 25 specifically expressed genes in each locus for each cell type. Finally, an enrichment scoring matrix was generated using the “get enrichment matrix” and “enrichment analysis” functions in the “Cottrazm” package. The enrichment scores for each cell type were visualized using the “SpatialFeaturePlot” function in the “Seurat” package. The malignant group was defined if the score of malignant cells in the microregion was 1, it is defined as the non-malignant group otherwise. Spearman correlation between gene expression and cell content was visualized by the “linkET” package.

### Immunohistochemical staining

A tissue microarray containing tumor samples and adjacent tissues from 76 CRC patients was obtained from Aifang company (AF-CocSur2201, China). The pathological sections were first subjected to deparaffinization and rehydration. Antigen retrieval was then performed using EDTA buffer. Endogenous peroxidase activity was subsequently blocked with hydrogen peroxide, followed by blocking with 3% bovine serum albumin. Tissue sections were incubated overnight at 4 degrees with the primary antibody against DNASE1L3 (GTX114363, GeneTex, USA). A secondary antibody (GB23303, Servicebio, China) was applied and incubated at room temperature for 1 hour. Diaminobenzidine was used as the chromogen to visualize immunoreactivity, and hematoxylin was employed for nuclear counterstaining. Stained specimens were imaged and analyzed using a microscope (Model KF-FL-020, KFBIO, China). The percentage of positively stained cells was quantified using Visiopharm software.

### Extraction of human peripheral blood

SLE patients, CRC patients, and healthy volunteers were recruited from the First Affiliated Hospital of Zhengzhou University. Peripheral blood was collected and centrifuged to obtain plasma. Peripheral blood mononuclear cells (PBMC) were extracted by density gradient centrifugation method using Lymphoprep reagent (1858, Serumwerk Bernburg AG, Germany).

### Cell culture

The human monocyte cell line THP-1, immortalized colon epithelial cell line NCM460, and the embryonic kidney cell line HEK-293T were purchased from HyCyte Company (China). THP-1, NCM460 and PBMC were cultured in RPMI 1640 medium (11875093, Gibco, USA) containing 10% fetal bovine serum (FBS, A5256701, Gibco, USA) and 1% penicillin/streptomycin (P/S, 15140122, Gibco, USA). HEK-293T cells were cultured in DMEM medium (11965092, Gibco, USA) containing FBS and P/S. All cells were cultured in 37 degrees incubator containing 5% CO2.

### Plasmid transfection

The DNASE1L3-targeting plasmids (sh-DNASE1L3), control plasmid (sh-NC), and helper plasmids (pSPAX2 and pMD2G) were synthesized by Tsingke company (China). These plasmids were transfected into HEK-293T cells using polyethylenimine (PEI; 40816ES01, Yeasen, China). After 48–72 hours of incubation, lentivirus-containing supernatants were harvested. THP-1 cells were then incubated with the lentiviral supernatant supplemented with 2 μg/mL polybrene (C0351, Beyotime, China) for 48 hours. Stable transductants were selected using 3 μg/mL puromycin (P8230, Solarbio, China). Successful knockdown of DNASE1L3 was confirmed by reverse transcription-quantitative polymerase chain reaction (RT-qPCR) and Western blotting analysis.

### RT-qPCR

Total RNA of cells was extracted by Trizol reagent (15596026CN, Thermofisher Scientific, USA). Then, RNA was reverse transcribed to cDNA according to the instructions of a Reverse Tran scription Kit (R433, Vazyme, China). A total of 15 pairs of cDNA microarrays of CRC tumor and matched paraneoplastic tissues were obtained from Shanghai Outdo Biotech Company (MecDNA-HColA030CS03, China). Next, qPCR was performed according to the instructions of a SYBR Green PCR Kit (Q712, Vazyme, China). Finally, the mRNA expression was obtained by the 2^-ΔΔCt^ method and standardized β-actin as the internal control. Primer sequences were listed in **Supplementary Table S1**.

### Enzyme-linked immunosorbent assay (ELISA)

Plasma DNASE1L3 levels in patients with SLE (n = 19) or CRC (n = 19), and healthy controls (n = 19) were quantified using an ELISA kit (CSB-EL007052HU, CUSABIO, China) according to the manufacturer’s protocol. Optical density at 450 nm was immediately measured using a Synergy H1 microplate reader (BioTek Instruments, USA).

### Western blotting

Cells were lysed on ice for 30 minutes in RIPA buffer (CW2333S, CWBIO, China) supplemented with 1% protease (CW2200S, CWBIO, China) and phosphatase inhibitor cocktail (CW2383S, CWBIO, China). Protein concentration was determined using a BCA assay kit (23227, Thermo Fisher Scientific, USA). Proteins were separated by SDS-PAGE and subsequently transferred onto PVDF membranes (IPVH00010, Millipore, USA). After blocking with 5% non-fat milk for 1 hour, membranes were incubated overnight at 4°C with primary antibodies against DNASE1L3 or β-tubulin (80762-1-RR, Proteintech, China). Next, membranes were probed with HRP-conjugated secondary antibodies (GAR1007, MultiSciences, China) for 1 hour at room temperature. Protein signals were detected using chemiluminescent reagent (GK10008, GLPBIO, USA). Relative expression levels of DNASE1L3 normalized to β-tubulin were quantified using ImageJ software.

### Efferocytosis assay

The NCM460 cells were labeled with 1 μM CMFDA (HY-126561, MedChemExpress, USA) for 30 minutes and then washed with PBS before being resuspended in complete culture medium. Apoptosis was induced in NCM460 cells by stimulation with 10 μM staurosporine (HY-15141, MedChemExpress, USA) for 12 hours, followed by washing with PBS. Transfected THP-1 cells were differentiated into macrophages by incubation with 100 ng/mL phorbol myristate acetate (PMA; HY-18739, MedChemExpress, USA) for 48 hours, as previously reported (37, 38). THP-1-derived macrophages were stained with 2 μM DiI (HY-126561, MedChemExpress, USA) for 20 minutes and washed with PBS. Labeled NCM460 cells and macrophages were co-cultured at a ratio of 5:1 for 4 hours. Non-phagocytosed and unbound apoptotic cells were then removed by washing with PBS. Total RNA was extracted for RT-qPCR analysis. Fluorescence signals were acquired on a FACS Canto II flow cytometer (BD Biosciences, USA). Data analysis was performed using FlowJo software (10.8.1, USA). Efferocytosis was quantified as the proportion of macrophages that had phagocytosed NCM460 cells, represented by the percentage of FITC-positive cells.

### Statistical analysis

All expression data from public databases was processed in R software (version 4.2.0). Unpaired t-tests were employed to compare two groups of variables exhibiting normal distributions. For non-normally distributed metrics, comparisons were made using the Mann-Whitney U test. Comparisons among multiple groups were performed using the Kruskal-Wallis test. Survival differences were assessed utilizing the log-rank test and visualized via Kaplan-Meier survival curves. The correlation was analyzed using the Spearman method.

## Results

### Identification of 58 common genes associated with CRC and SLE

**Figure 1:**
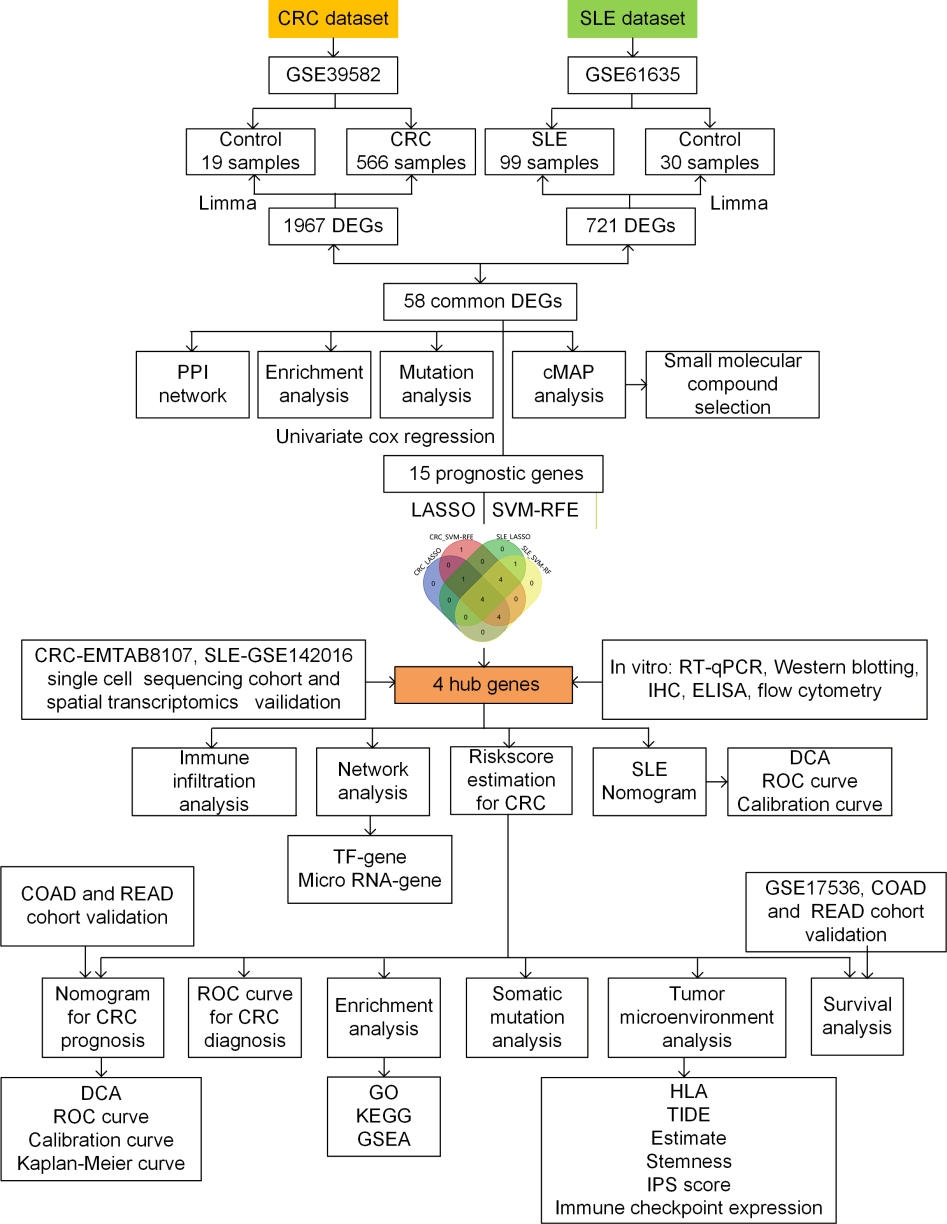
Flow chart of the study design. TF, transcription factors; CRC, colorectal cancer; SLE, systemic lupus erythematosus; DEG, differentially expressed gene; PPI, protein-to-protein interaction; cMAP, connectivity map; LASSO, least absolute shrinkage and selection operator; SVM-RFE, support vector machine-recursive feature elimination; RT-qPCR, reverse transcription-quantitative polymerase chain reaction; ELISA, enzyme-linked immunosorbent assay; IHC, immunohistochemical; ROC, receiver operating characteristic; COAD, colon adenocarcinoma; READ, rectum adenocarcinoma; DCA, decision curve analysis; GO, Gene Ontolog; KEGG, Kyoto Encyclopedia of Genes and Genomes; GSEA, gene set enrichment analysi; HLA, human leukocyte antigen; TIDE, tumor immune dysfunction and exclusion; IPS, immunophenotypic score.

**Figure 2:**
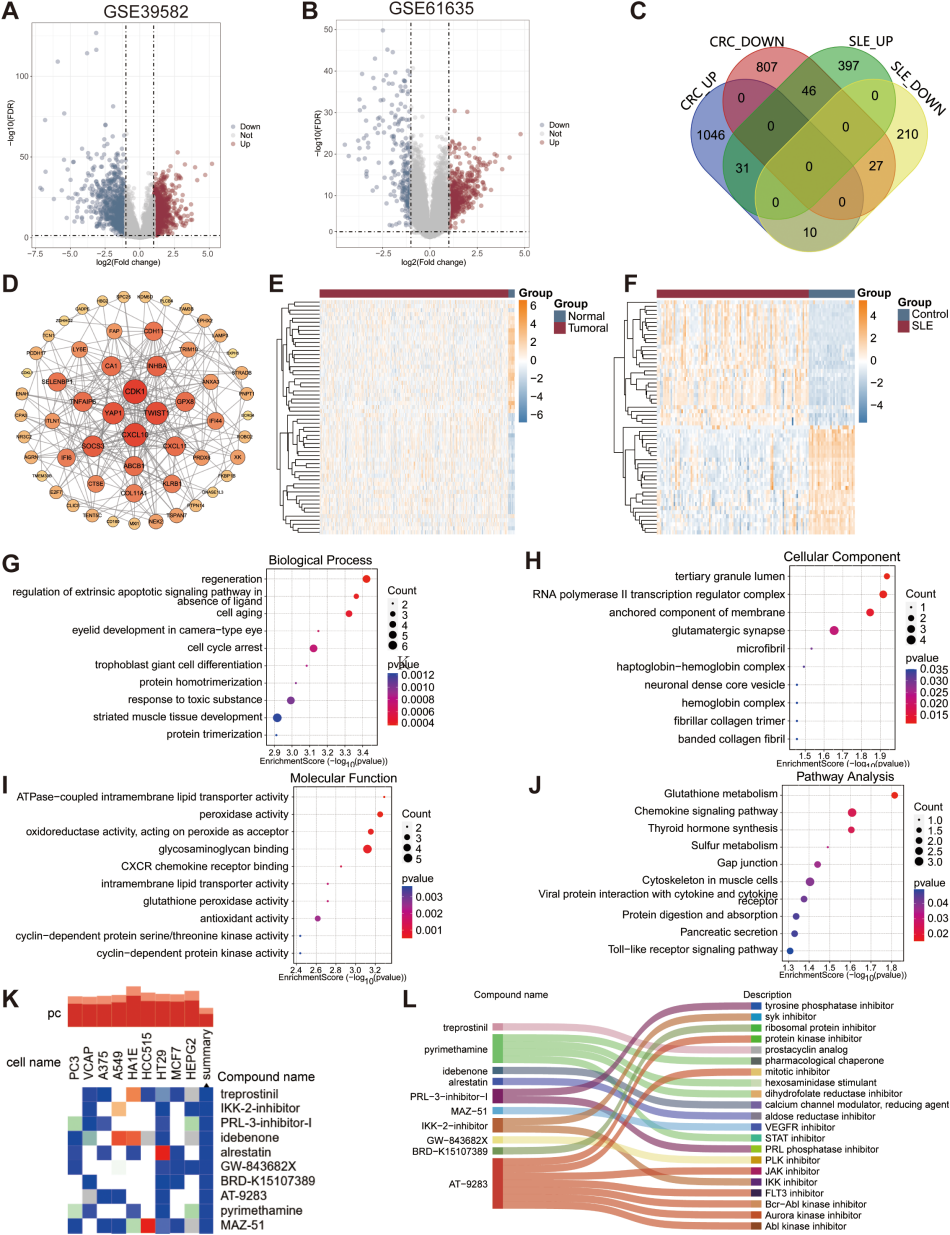
Identification of common DEGs associated with CRC and SLE. **(A)** The volcano plot representing 1967 DEGs in the CRC-GSE39582 dataset. Red dots were represented 1087 upregulated genes and blue dots were represented 880 downregulated genes. **(B)** The volcano plot showing 721 DEGs in the SLE-GSE61635 dataset. Red dots were represented 474 up-regulated genes and blue dots were represented 247 down-regulated genes. **(C)** The Venn diagram displayed 58 common DEGs of CRC and SLE cohorts. **(D)** The PPI network revealing the interactions among common DEGs. Node size and color reflect the degree of connectivity. The heatmaps representing the expression pattern of common DEGs in the GSE39582 **(E)** and GSE61635 **(F)** dataset. The color scale reflects standardized expression values (Z-scores), with red indicating higher expression and blue indicating lower expression. GO-biological processs **(G)**, GO-cellular component **(H)**, GO-molecular function **(I)**, and KEGG **(J)** enrichment analyses of common DEGs. **(K)** Heatmap depicting the top 10 compounds with the most significant negative enrichment scores based on cMAP database. The color scale represents the enrichment score (red, positive; blue, negative). **(L)** Sankey diagram illustrating functional annotations of top 10 compounds.

The strategy of data processing was shown in **Figure 1**. Differential analysis showed 1967 DEGs between CRC and control tissues in the GSE39582 cohort, including 1087 up-regulated genes and 880 down-regulated genes. In the GSE61635 cohort, 721 DEGs, including 474 up-regulated genes and 247 down-regulated genes, were identified in PBMCs from SLE and controls. Volcano plots were applied to demonstrate the expression pattern of DEGs in GSE39582 and GSE61635 datasets (**Figures 2A, B**). To further explore the shared key genes of CRC and SLE, Venn diagrams were used to showed the 58 common DEGs of CRC and SLE (**Figure 2C**). The protein-to-protein interaction (PPI) network illustrated the interactions among proteins associated with the common DEGs (**Figure 2D**). Heatmaps were carried out to identify the expression pattern of common DEGs in GSE39582 and GSE61635 cohorts (**Figures 2E, F**). Biological process of GO analysis revealed that the common DEGs were enriched in regeneration, regulation of extrinsic apoptotic signaling pathway in absence of ligand, and cell aging (**Figure 2G**). Cellular component of GO analysis showed that the DEGs were enriched in tertiary granule lumen, RNA polymerase II transcription regulator complex, and anchored component of membrane (**Figure 2H**). Molecular function of GO analysis displayed that the DEGs were enriched in ATPase-coupled intramembrane lipid transporter activity, peroxidase activity, and oxidoreductase activity, acting on peroxide as acceptor (**Figure 2I**). KEGG analysis demonstrated that the DEGs were enriched in glutathione metabolism, chemokine signaling pathway, and thyroid hormone synthesis (**Figure 2J**).

### Screening of ten candidate small-molecule compound for treatment

The 31 up-regulated DEGs were uploaded to the cMap database to identify potential therapeutic drugs. After significance screening, the top 10 compounds—including treprostinil, IKK-2-inhibitor, PRL-3-inhibitor-I, idebenone, alrestatin, GW-843682X, BRD-K15107389, AT-9283, pyrimethamine, and MAZ-51, were identified as potential therapeutic agents for CRC and SLE (**Figure 2K**). These compounds primarily function as phosphatase inhibitors, VEGFR inhibitors, calcium channel modulators, etc. (**Figure 2L**). The chemical structures of these 10 compounds are shown in **Supplementary Figure S1**.

### Identification of four hub genes in CRC and SLE

**Figure 3:**
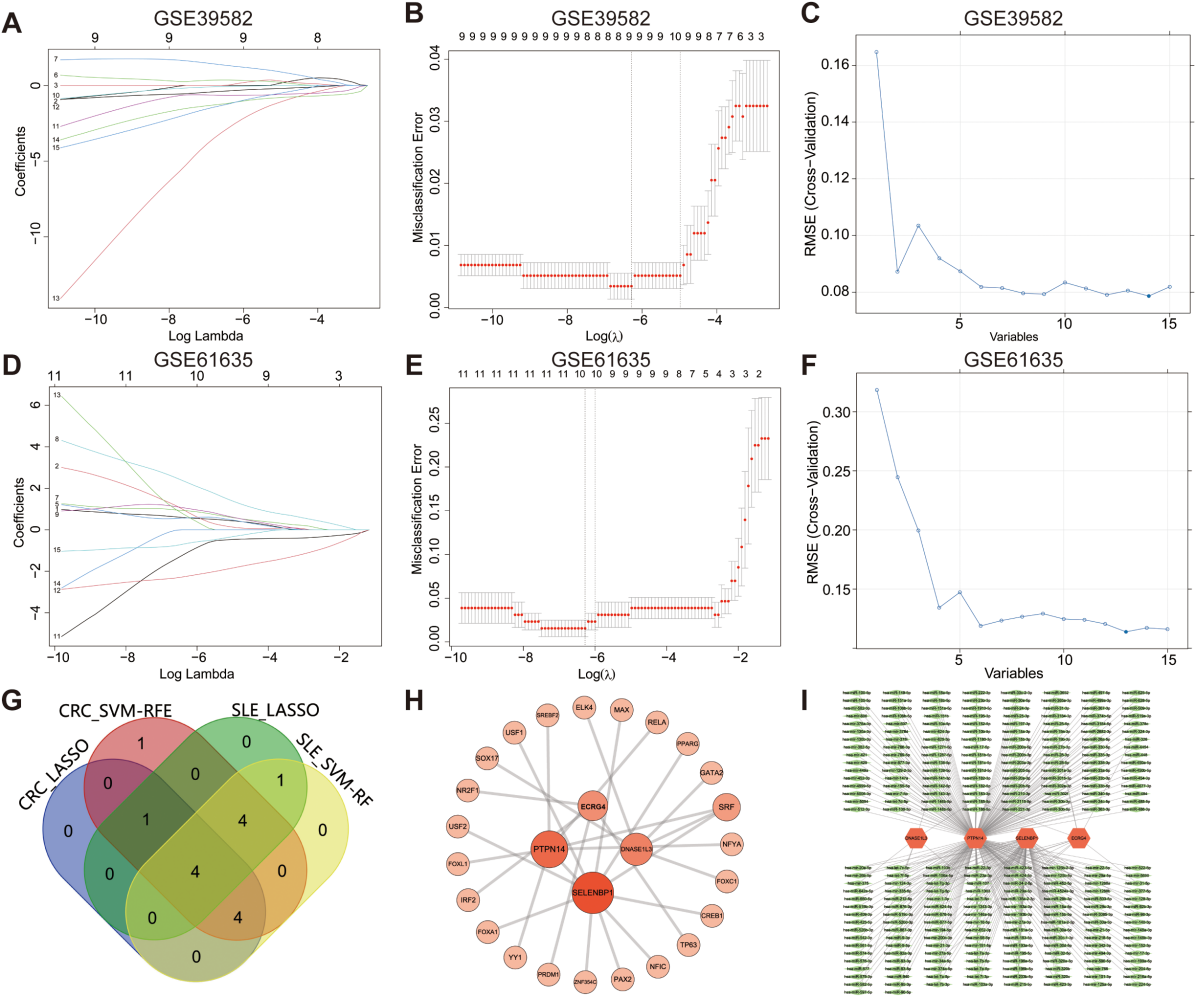
Identification of potential biomarkers for CRC and SLE via machine learning methods. The coefficient profile plot **(A)** and misclassification errors plot **(B)** of LASSO algorithm representing selected nine variables for CRC. **(C)** The SVM-RFE algorithm displaying selected 14 genes for CRC. The coefficient profile plot **(D)** and misclassification errors plot **(E)** of LASSO algorithm displaying selected 10 genes for SLE. **(F)** The SVM-RFE algorithm displaying selected 13 genes for SLE. **(G)** The Venn diagram displaying four common genes, which were identified as the diagnostic biomarkers for CRC and SLE. **(H)** Interaction between four hub genes and transcription factors. **(I)** Interaction between four hub gene and microRNAs. Edge lines indicate experimentally supported regulatory interactions.

The univariate Cox, SVM-RFE, and LASSO were applied to explore the hub genes in CRC and SLE. First, the univariate Cox regression was carried out to identify 15 potential candidate genes from 58 common DEGs. The LASSO regression was used to identify 9 potential genes from CRC and 10 genes from SLE. The SVM-RFE algorithm was constructed to identified 14 potential genes from CRC and 13 potential genes from SLE (**Figures 3A–F**). The overlapping genes among all subsets were defined as hub genes, i.e., genes associated with SLE diagnosis as well as the diagnosis and prognosis of CRC. A Venn diagram identified the four hub genes—*DNASE1L3*, protein tyrosine phosphatase non-receptor type 14 (*PTPN14*), selenium-binding protein 1 (*SELENBP1*), and esophageal cancer related gene 4 (*ECRG4*)—which were subsequently included in further analyses (**Figure 3G**). Network analysis via the NetworkAnalyst database revealed that the four signature genes are regulated by transcription factors such as SRF, YY1, and IRF2, as well as miRNAs including hsa-mir-335-5p, hsa-mir-27a-3p, and hsa-mir-124-3p (**Figures 3H, I**).

### Partially overlapping immune infiltration patterns of CRC and SLE

**Figure 4:**
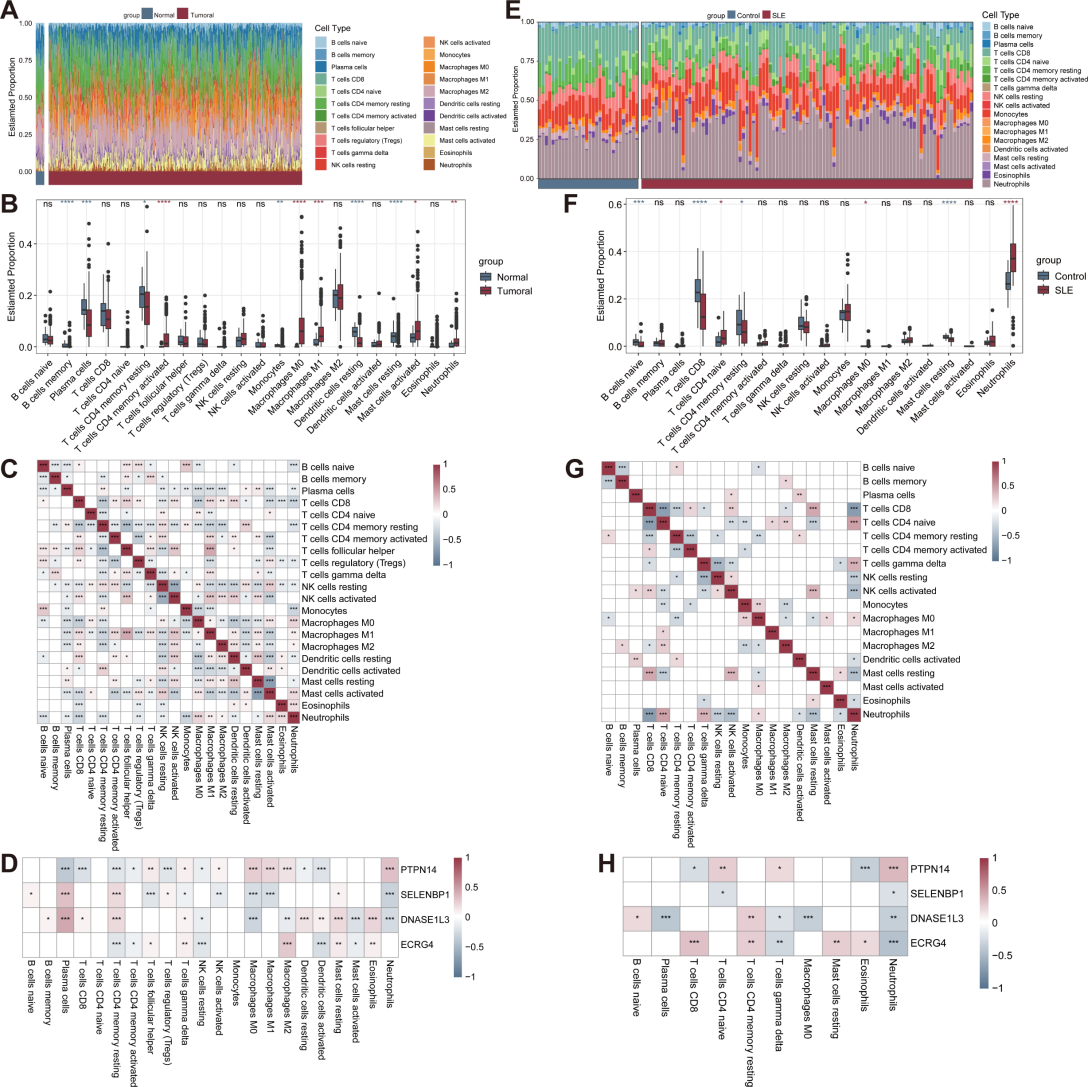
Analysis of immune cell infiltration via the CIBERSORT algorithm. **(A)** Stacked bar plot showing the proportions of immune cells in tumor versus control group in the GSE39582 cohort. **(B)** Comparison of 22 immune cell types between the tumor and the control group. **(C)** Heatmap showing the correlations among 22 immune cell types in tumor samples. **(D)** Heatmap revealing the associations between four genes and 22 immune cell types in tumor samples. **(E)** Stacked bar plot showing the proportions of immune cells in the SLE versus control group in the GSE61635 cohort. **(F)** Comparison of 22 immune cell types between the SLE and control group. **(G)** Heatmap displaying the correlations among 22 immune cell types in SLE samples. **(H)** Heatmap revealing the associations between 4 genes and 22 immune cell types in SLE samples. *p < 0.05; **p < 0.01; ***p < 0.001; ****p < 0.0001; ns not significant. CIBERSORT, cell-type identification by estimating relative subsets of RNA transcripts.

The proportion of immune cells in each sample of CRC cohort was calculated by the “CIBERSORT”algorithm (**Figure 4A**). There were significant differences in the proportions of the 11 types of immune cells between the control and CRC groups. Compared to the control group, the CRC group had a higher proportion of M0 macrophages, M1 macrophages, activated mast cells, neutrophils, activated memory CD4 T cells, while a lower proportion of memory B cells, resting dendritic cells, resting mast cells, monocytes, plasma cells, and resting memory CD4 T cells (**Figure 4B**). In addition, correlation analysis of immune cells showed a significant positive correlation between M1 macrophages and follicular helper T cells (r = 0.449, P < 0.001), and a significant negative correlation between activated NK cells and resting NK cells (r = -0.566, P < 0.001) (**Figure 4C**). Moreover, the correlation revealed that the four genes were closely associated with immune cell infiltration. Plasma cells were positively correlated with SELENBP1, and DNASE1L3. Neutrophils were negatively correlated with SELENBP1 (**Figure 4D**).

Similarly, the proportion of immune cells in each sample of SLE cohort was displayed in **Figure 4E**. There were significant differences in the proportions of the 7 types of immune cells between the control and SLE groups. Compared to the control group, the SLE group had a higher proportion of M0 macrophages, neutrophils, yet a lower proportion of naive B cells, resting mast cells, resting memory CD4 T cells, and CD8 T cells (**Figure 4F**). There was a significant positive correlation between neutrophils and naive CD4 T cells (r = 0.481, P < 0.001), and a significant negative correlation between neutrophils and CD8 T cells (r = -0.695, P < 0.001) (**Figure 4G**). PTPN14 expression showed the highest positive correlation with neutrophil infiltration and highest negative correlation with eosinophil infiltration. SELENBP1 expression showed the highest negative correlation with neutrophils. DNASE1L3 expression demonstrated the strongest positive correlation with resting memory CD4T cells and the strongest negative correlation with plasma cells. ECRG4 expression shows the highest positive correlation with CD8 T cells, while it shows the highest negative correlation with neutrophils (**Figure 4H**).

### Diagnostic efficacy of the nomogram model for SLE

**Figure 5:**
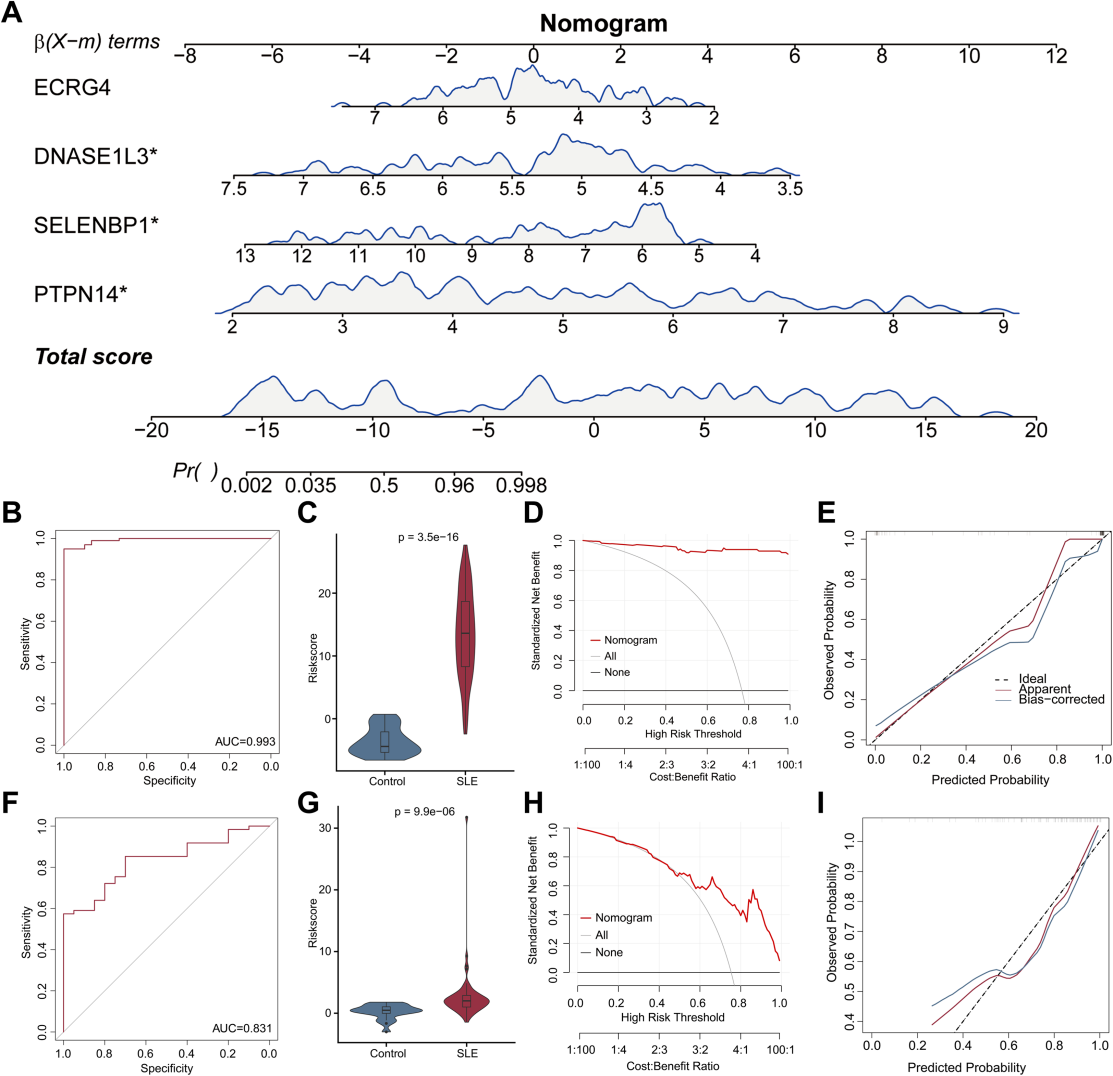
Construction and evaluation of the diagnostic nomogram model for SLE. **(A)** Construction of the diagnostic nomogram model for SLE in the GSE61635 dataset using four genes. Each gene is assigned a point value on the “Points” scale. The total points correspond to a predicted probability of SLE on the bottom scales. **(B)** The ROC curve revealed an AUC of 0.993 for the nomogram model for diagnosing SLE. **(C)** The violin plot displaying significantly higher risk score for SLE compared to controls. **(D)** The DCA curve for the nomogram model. The x−axis represents threshold probability, and the y−axis represents net benefit. Curves for the nomogram, “treat all,” and “treat none” strategies are shown, indicating the clinical utility of the model across a range of decision thresholds. **(E)** The calibration curve showing that the predicted and observed probabilities of the nomogram model. The ROC **(F)**, violin **(G)**, DCA **(H)**, calibration **(I)** curves of the nomogram model for diagnosing SLE in the GSE50772 dataset.

In order to diagnose and predict SLE, four signature genes were used to construct a diagnostic nomogram model of SLE with the “rms” package (**Figure 5A**). ROC curves were applied to calculate the area under curve (AUC)values of the nomogram model to determine their sensitivity and specificity for diagnosing SLE. The results showed that the nomogram model had an AUC value of 0.993 for the diagnosis of SLE (**Figure 5B**). Based on the nomogram model, risk scores were calculated for each sample, and the violin plot demonstrated significantly higher risk scores for SLE compared to the control group (**Figure 5C**). Additionally, DCA calculated the clinical “net benefit” of the nomogram model at different threshold probabilities compared to a strategy of treating all patients or no patients (**Figure 5D**). Furthermore, the calibration plot revealed the predicted probabilities of the nomogram model were almost identical to the actual probabilities (**Figure 5E**). Similarly, the model showed good performance in discriminating SLE patients from controls in the GSE50772 dataset (**Figures 5F–I**).

### Four-gene-based risk score for the diagnosis and prognostic prediction of CRC

**Figure 6:**
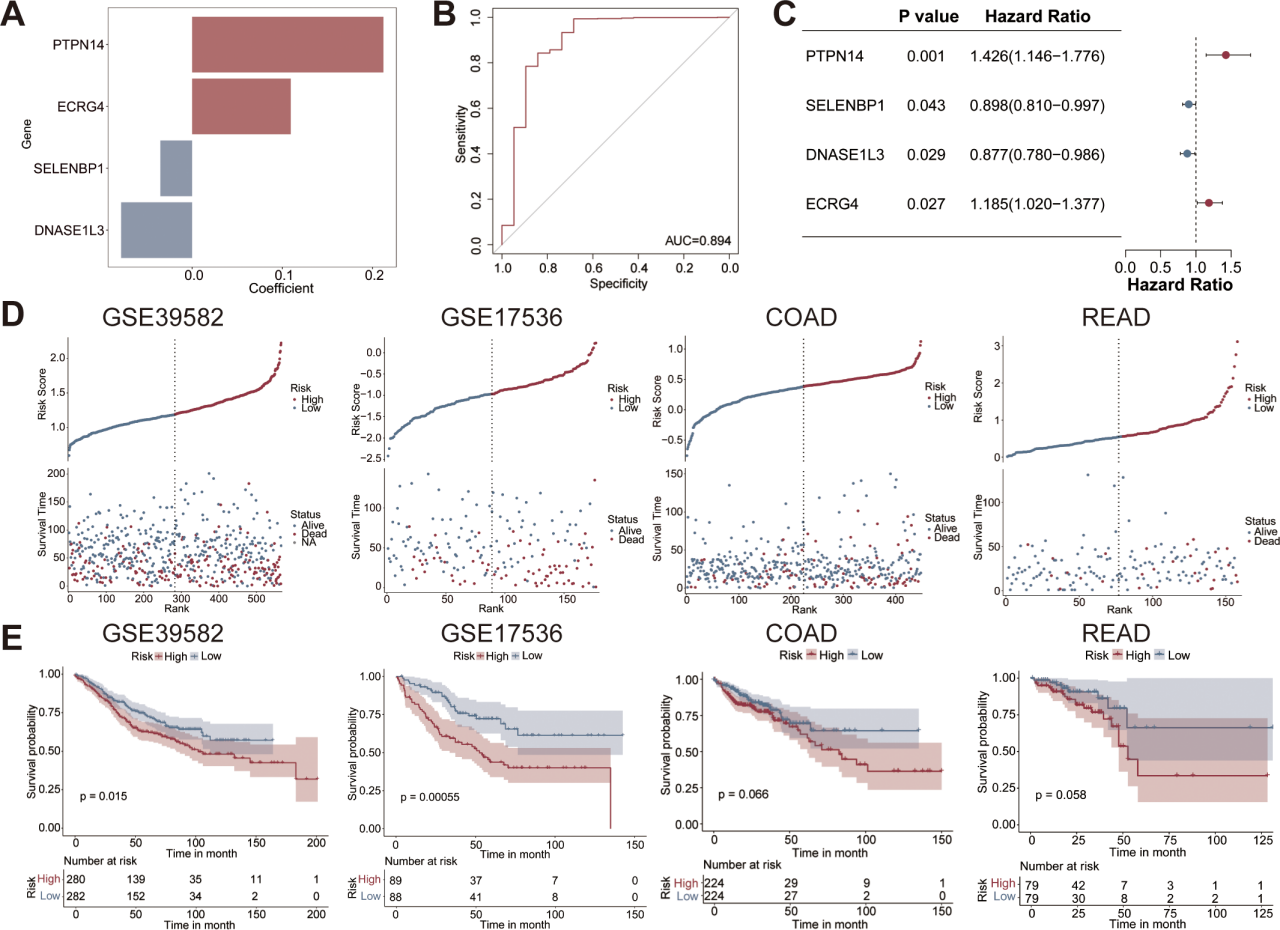
Construction of a prognostic gene signature in CRC. **(A)** The four genes and their coefficients in the risk score. **(B)** ROC curve of the 4-gene-based risk score for diagnosing CRC. **(C)** Forest plot for predicting CRC prognosis based on the four hub genes. **(D)** Distribution of risk scores and survival status across the cohorts. **(E)** Kaplan-Meier survival curves for high-risk and low-risk groups in the GSE39582, GSE17536, COAD, and READ cohorts.

The risk score for each sample was calculated based on the expression levels of the four genes. The risk score was defined as follows: Risk score = (0.2120) × PTPN14 + (-0.0354) × SELENBP1 + (-0.0788) × DNASE1L3 + (0.1093) × ECRG4. The histogram displayed the coefficients of the four genes in the risk score model (**Figure 6A**). The AUC values for the risk score in diagnosing CRC were 0.894 in GSE39582 (**Figure 6B**), 0.691 in COAD (**Supplementary Figure S2A**), and 0.668 in READ (**Supplementary Figure S2B**). The forest plot indicated that the four gene significantly influenced the survival prognosis of CRC (**Figure 6C**). CRC patients were stratified into high and low risk groups based on the median value of risk score. The risk of death was increased in the high-risk group compared to the low-risk group (**Figure 6D**). Notably, survival analysis uncovered that OS was significantly lower in the high-risk group compared with the low-risk group in the GSE39582 and GSE17536 cohorts. Patients in the high-risk group demonstrated a trend toward reduced OS compared to those in the in the low-risk group in the COAD and READ cohorts (**Figure 6E**).

**Figure 7:**
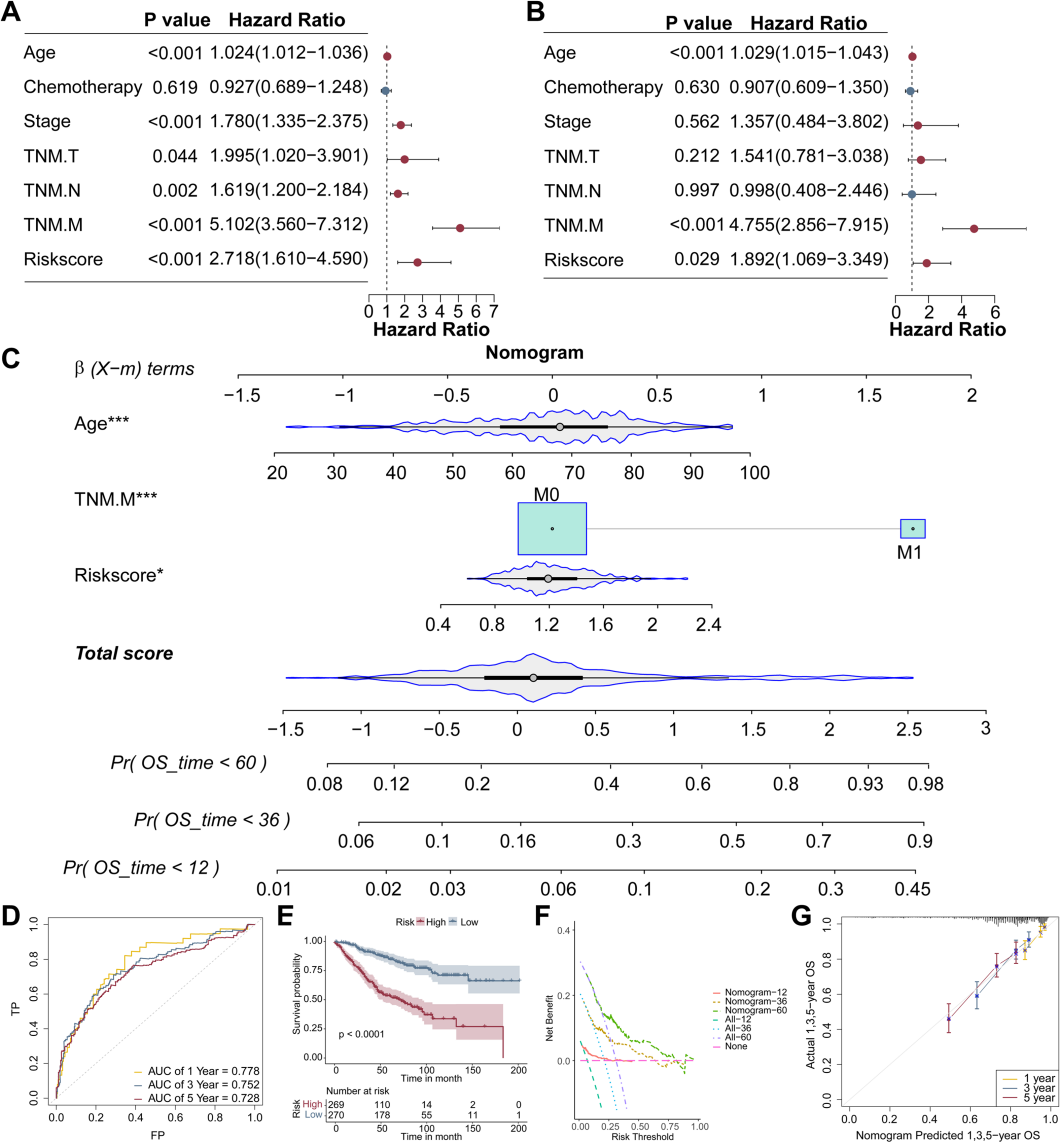
Construction and validation of prognostic nomogram model for CRC. Univariate Cox **(A)** and multivariate Cox **(B)** analysis revealing significant prognostic factors in the GSE39582 cohort. **(C)** Nomogram model based on age, M classification, and risk score for predicting survival. **(D)** ROC curve showing the AUC value of the nomogram model for predicting OS. **(E)** Kaplan-Meier survival curve comparing OS between the high-risk and low-risk groups. **(F)** DCA curve evaluating the clinical applicability of the nomogram model. **(G)** Calibration curve demonstrating the consistency between predicted and actual values. AUC, area under curve; OS, overall survival.

Risk score, age, chemotherapy, clinical stage, T classification, N classification, and M classification were included in univariate Cox regression, and the results revealed that risk score, age, clinical stage, T classification, N classification, and M classification significantly affected prognosis of patients (**Figure 7A**). Multivariate Cox regression analysis verified that risk score, age, and M classification were significant prognostic factors for patients (**Figure 7B**). The above results showed that risk score, age, and M classification were independent prognostic factors for CRC patients. The associations between the four hub genes and key clinicopathological features of CRC were further systematically analyzed. The results indicated that the expression levels of PTPN14 and ECRG4 were higher in patients with T3–4 classification (**Supplementary Figure S3A**). Additionally, PTPN14 expression was elevated in patients with N1–2 classification (**Supplementary Figure S3B**). We also observed that DNASE1L3 expression was lower in M0 patients, whereas PTPN14 expression was higher in M1 patients (**Supplementary Figure S3C**). Furthermore, PTPN14 levels were increased in patients with stage III–IV (**Supplementary Figure S3D**), and ECRG4 expression was higher in patients who received chemotherapy (**Supplementary Figure S3E**).

Based on the significant factors, a nomogram model was constructed to predict the prognosis of CRC (**Figure 7C**). The nomogram demonstrated AUC values of 0.778, 0.752, and 0.728 for predicting 1-, 3-, and 5-year survival of CRC, respectively (**Figure 7D**). Patients in the high-risk group demonstrated a significantly poorer prognosis compared to those in the low-risk group (**Figure 7E**). DCA showed that the clinical “net benefit” of the predictive model at different threshold probabilities was higher than that of the strategy of treating all patients or not treating patients (**Figure 7F**). Moreover, the calibration curve revealed minimal deviation between the actual and predicted probabilities (**Figure 7G**).

Similar results were obtained in the validation cohort. The nomogram model demonstrated time-dependent predictive accuracy for COAD, with AUC values of 0.752, 0.728, and 0.679 at 1, 3, and 5 years, respectively. Furthermore, survival, DCA, and calibration curves collectively validated its robust predictive performance (**Supplementary Figures S4A–D**). Due to the lack of sufficient data on the 5-year survival of patients in the READ cohort, we analyzed the accuracy of the nomogram model in predicting 1-, 2-, and 3-year survival for READ. The AUC values were 0.842, 0.802, and 0.806, respectively (**Supplementary Figure S4E**). Furthermore, survival, DCA, and calibration curves further validated the model’s robust predictive capability (**Supplementary Figures S4F–H**). These results suggested that the nomogram model had good diagnostic and prognostic prediction efficacy for CRC.

### Mutational profiles between high-risk and low-risk subgroups in CRC

**Figure 8:**
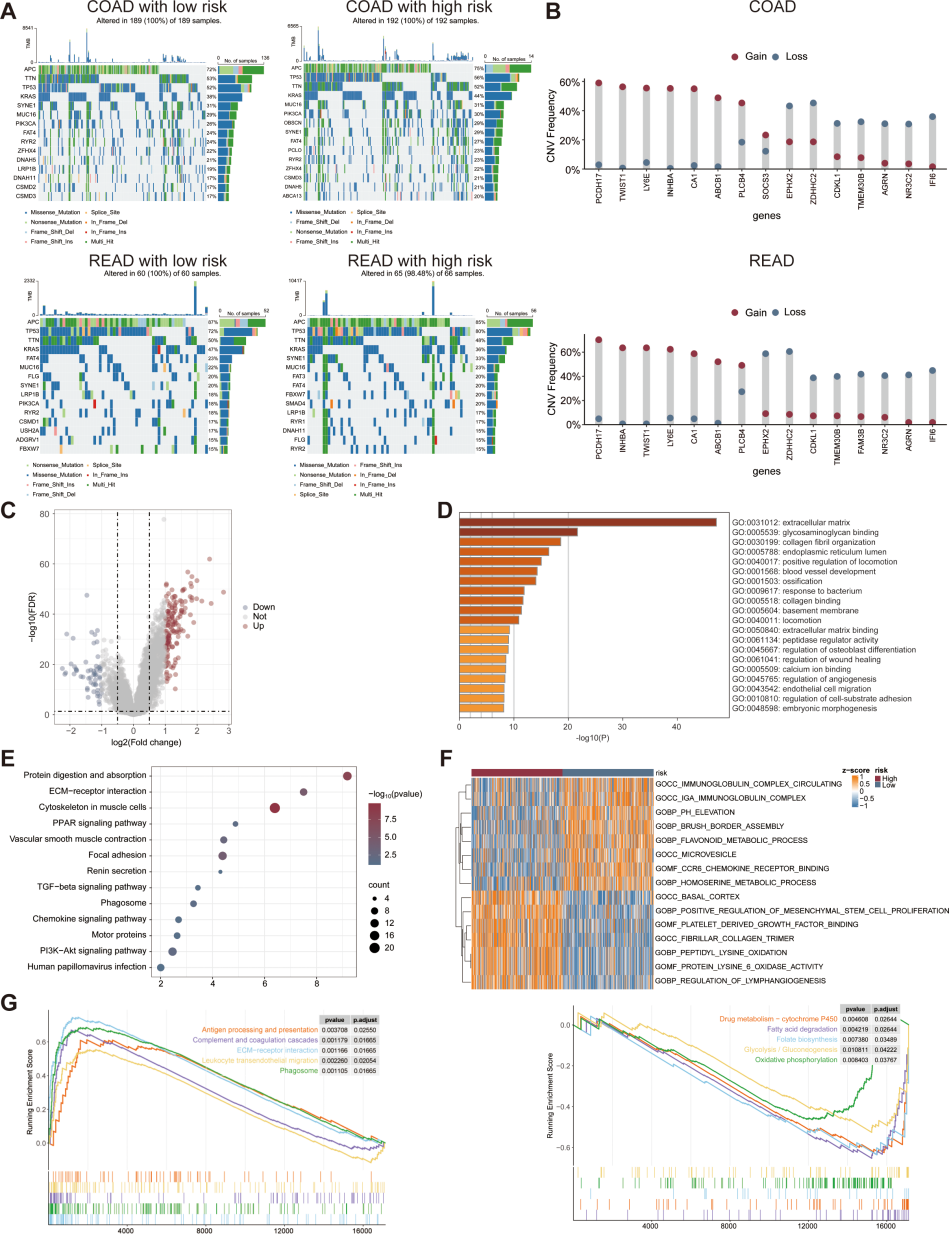
Mutation and functional analysis between high- and low-risk group in the CRC patients. **(A)** Differences in somatic mutation patterns of the top 15 genes across two risk cohorts in the COAD and READ cohort. **(B)** CNV frequency in COAD and READ cohorts. **(C)** The volcano plot representing 204 DEGs between two groups. GO **(D)**, KEGG **(E)**, GSVA **(F)**, and GSEA **(G)** Enrichment analysis of DEGs between two groups. CNV, copy number variation; GSVA, gene set variation analysis.

Somatic mutation analysis revealed that APC, TP53, and TTN were the three most frequently mutated genes in both high-risk and low-risk groups across the COAD and READ cohorts (**Figure 8A**). In the COAD cohort, copy number amplifications were predominantly observed in PCDH17, TWIST1, and LY6E genes, whereas in the READ cohort, amplifications were more commonly detected in PCDH17, INHBA, and TWIST1. Copy number deletions were consistently prevalent in ZDHHC2, EPHX2, and IFI6 genes in both cohorts (**Figure 8B**).

### Distinct biological mechanisms drive tumor progression between high-risk and low-risk groups in CRC

A total of 204 DEGs were identified between the low- and high-risk group in the GSE39582 cohort. The distribution of these DEGs was shown in the volcano plot (**Figure 8C**). To gain a better understanding of potential molecular processes and functions, GO, KEGG, GSVA, and GSEA enrichment analysis was then conducted based on the DEGs. GO analysis illustrated that pathogenic genes were closely enriched in extracellular matrix, glycosaminoglycan binding, and collagen fibril organization (**Figure 8D**). KEGG analysis revealed that pathogenic genes were mostly enriched in protein digestion and absorption, ECM-receptor interaction, and cytoskeleton in muscle cells (**Figure 8E**). GSVA analysis indicated that DEGs were mostly clustered in immunoglobulin complex circulating, IgA immunoglobulin complex, and pH elevation (**Figure 8F**). GSEA analysis revealed that the antigen processing and presentation, complement and coagulation cascades, leukocyte transendothelial migration, ECM-receptor interaction, and phagosome were enriched in the high-risk group, while drug metabolism cytochrome P450, fatty acid degradation, folate biosynthesis, glycolysis/gluconeogenesis, and oxidative phosphorylation were enriched in the low-risk group (**Figure 8G**).

### Different immune response patterns between high- and low-risk groups in CRC

**Figure 9:**
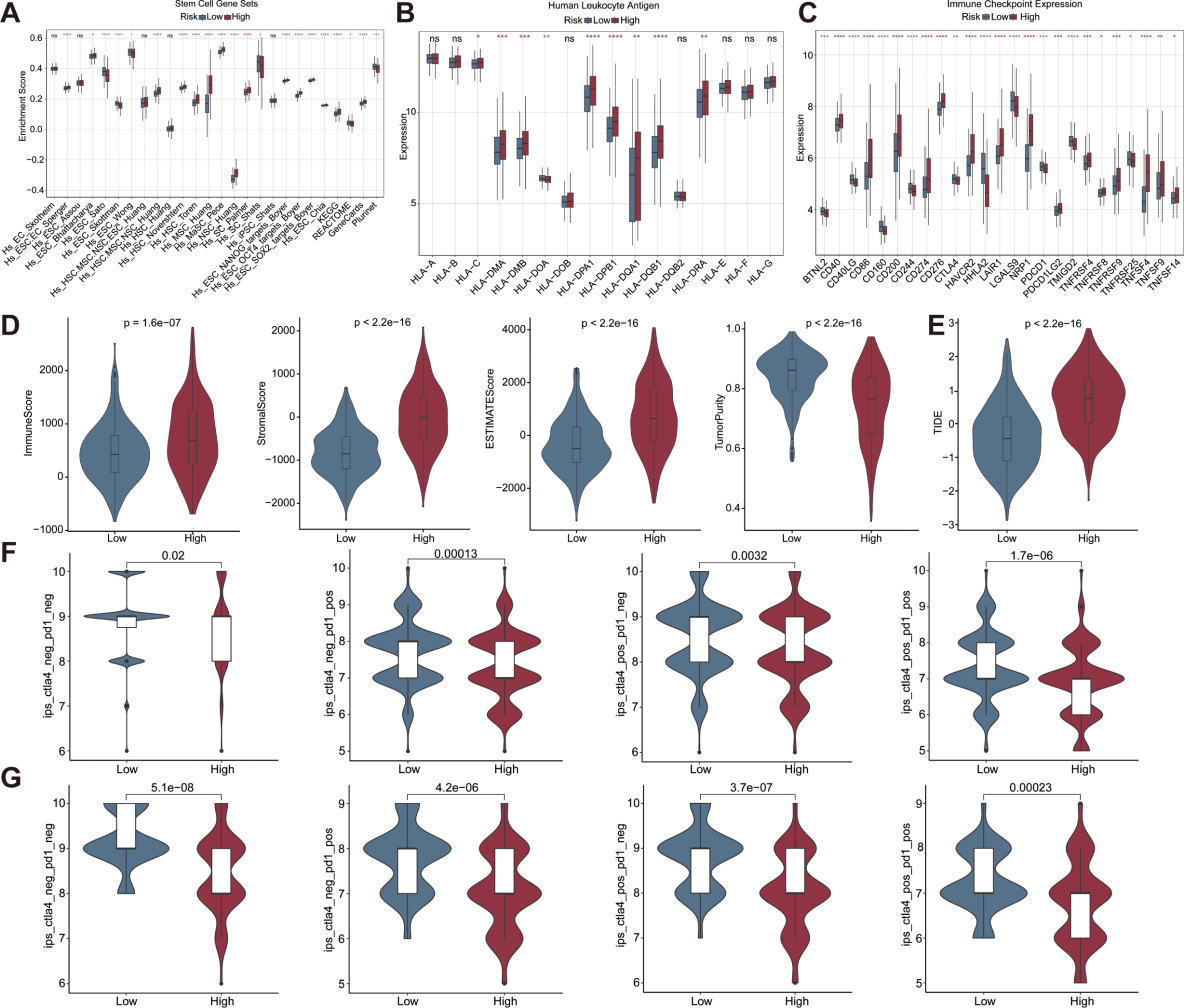
Differences in immune response patterns between high and low risk groups in the CRC datasets. Differences in the stemness enrichment scores **(A)**, immune checkpoint gene expression **(B)**, HLA typing **(C)**, immune score, stroma score, ESTIMATE score, tumor purity **(D)**, TIDE score **(E)** between the high- and low-risk groups. IPS score across two risk cohorts in the COAD **(F)** and READ **(G)** cohort. ESTIMATE, estimation of stromal and immune cells in malignant tumor tissues using expression data.

Cancer stem cells are a very small number of tumor cells in tumor tissue with biological properties including unlimited proliferation, self-renewal and multidirectional differentiation. There was a significant difference in stemness enrichment scores between the high- and low-risk groups by 26 stemness gene sets, suggesting that the pattern of tumor genesis, progression, infiltration and recurrence after treatment may be different between the two groups (**Figure 9A**).

Functional enrichment analysis of signature genes showed a close association with immune cell infiltration in CRC. Therefore, we explored the correlation between risk score and immune cell characteristics. Analyses of HLA typing showed significant differences in the 9 HLAs between the high- and low-risk groups. Eight HLA were upregulated in the high-risk group, whereas only one HLA was upregulated in the low-risk group (**Figure 9B**). The expression levels of CD40, CD86, CD200, CD274, CD276, HAVCR2, LAIR1, NRP1, PDCD1LG2, TNFRSF4, TNFRSF8, TNFRSF9, TNFSF4, TNFSF9, and TNFSF14 were significantly higher in the high-risk group, while BTNL2, CD40LG, CD160, CD244, CTLA4, HHLA2, LGALS9, PDCD1, TMIGD2, and TNFRSF25 were lower (**Figure 9C**). Differences in immune checkpoint gene expression between high- and low-risk groups may result in differential susceptibility to immune checkpoint inhibitors. Furthermore, analysis using the estimation of stromal and immune cells in malignant tumor tissues using expression data (ESTIMATE) algorithm revealed that the high-risk group exhibited significantly higher immune, stromal, and ESTIMATE scores, along with lower tumor purity (**Figure 9D**). In addition, TIDE score in high-risk patients was significantly higher than that of low-risk patients, suggesting a worse efficacy of immunotherapy (**Figure 9E**). Finally, we compared the IPS between the two risk groups in COAD and READ cohorts. The low-risk group exhibited a significantly elevated IPS, indicating a more promising potential for immune response. In contrast, the high-risk group showed a markedly reduced IPS, suggesting a potential resistance to immune checkpoint inhibitors (**Figures 9F, G**). In conclusion, there were significant differences in the microenvironment between high and low risk patients. These heterogeneities may contribute to more precise tumor immunotherapy.

### Single-cell analysis revealed expression and functional profiles of four genes in CRC and SLE

**Figure 10:**
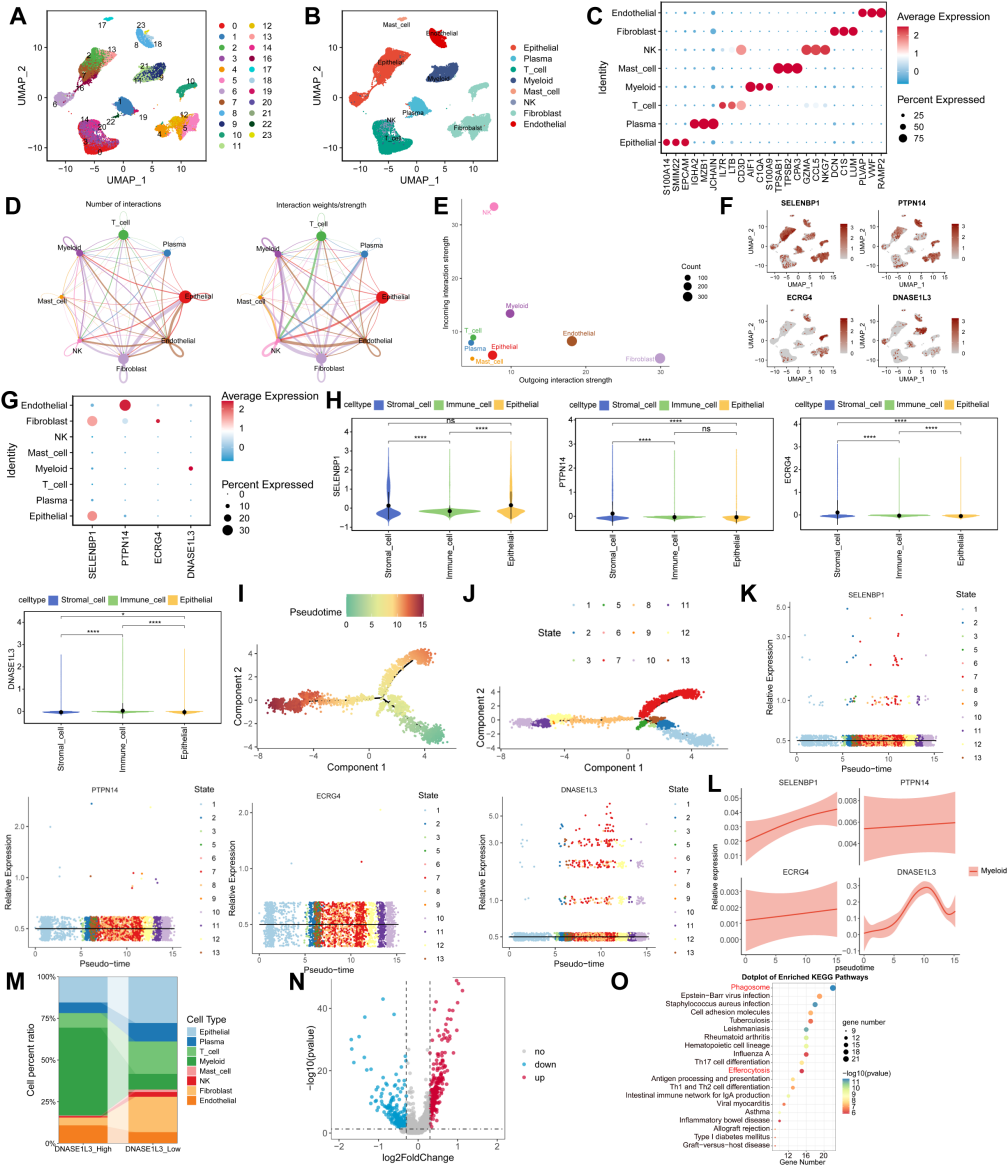
Single-cell analysis reveals expression and functional characteristics of four genes in CRC in the E-MTAB-8107 dataset. **(A)** UMAP plot showing 23 clusters identified. **(B)** UMAP visualization annotated with 8 major cell types. **(C)** Heatmap exhibiting the top 3 marker genes for each cell type. **(D)** Analysis of intercellular communication networks. **(E)** Bar plot or circle plot identifying major “sender” (signal-emitting) and “receiver” (signal-receiving) cell types based on cumulative outgoing and incoming signaling strengths. **(F)** UMAP plots displaying the distribution and expression levels of the four genes across 8 cell types. **(G)** Heatmap illustrating expression patterns of the four genes in the 8 cell types. **(H)** Violin plots showing expression levels of the four genes across three broad cellular categories: immune cells, stromal cells, and epithelial cells. **(I)** Pseudotime trajectory reconstruction. Each point represents a single cell; colors indicate pseudotime progression from early to late states along inferred lineages. **(J)** Cell state distribution along pseudotime. **(K)** Developmental trajectories of the four genes across pseudotime. **(L)** Dynamic expression changes of the four genes along pseudotime. **(M)** Cellular composition analysis comparing DNASE1L3-high and DNASE1L3-low expression groups. **(N)** Volcano plot displaying 360 DEGs between the two groups. **(O)** KEGG pathway enrichment analysis of identified DEGs. *p < 0.05; ****p < 0.0001; ns, not significant; UMAP, uniform manifold approximation and projection.

Analysis of single-cell RNA sequencing data from 7 CRC patients in the E-MTAB-8107 dataset identified 23 clusters, which were further classified into 8 distinct cell types, including epithelial cells, plasma cells, T cells, myeloid cells, mast cells, NK cells, fibroblast cells, and endothelial cells (**Figures 10A, B**). The top three marker genes for each cell type are shown in **Figure 10C**. Cell communication analysis revealed that NK cells were the primary signal receivers, while fibroblast cells were the major signal senders (**Figures 10D, E**, **Supplementary Figure S5**). UMAP visualization illustrated the distribution and expression levels of the four feature genes across the eight cell types (**Figure 10F**). These findings indicate that DNASE1L3 was predominantly expressed in myeloid cells, PTPN14 in endothelial cells, SELENBP1 in epithelial and fibroblast cells, and ECRG4 mainly in fibroblastic cells (**Figure 10G**). The eight cell types were categorized into three broad classes: immune cells, epithelial cells, and stromal cells. We observed that DNASE1L3 was primarily distributed in immune cells, PTPN14 in stromal cells, SELENBP1 in both epithelial and stromal cells, and ECRG4 mainly in stromal cells (**Figure 10H**). Pseudotime analysis was subsequently performed (**Figure 10I, J**), revealing distinct developmental patterns for the four genes (**Figure 10K**). The expression of PTPN14, SELENBP1, and ECRG4 gradually increased along the pseudotime trajectory, whereas DNASE1L3 expression initially increased and then decreased (**Figure 10L**). Furthermore, all cells were stratified into two subgroups based on DNASE1L3 expression status (high *vs*. low) to evaluate the contribution of different cell types to gene expression. Myeloid cells were identified as the primary contributors to DNASE1L3-high expression (**Figure 10M**). A total of 360 DEGs were identified between DNASE1L3- high and - low cells, as shown in **Figure 10N**. KEGG enrichment analysis indicated that these DEGs were significantly enriched in pathways such as phagosome, Epstein–Barr virus infection, and staphylococcus aureus infection (**Figure 10O**).

**Figure 11:**
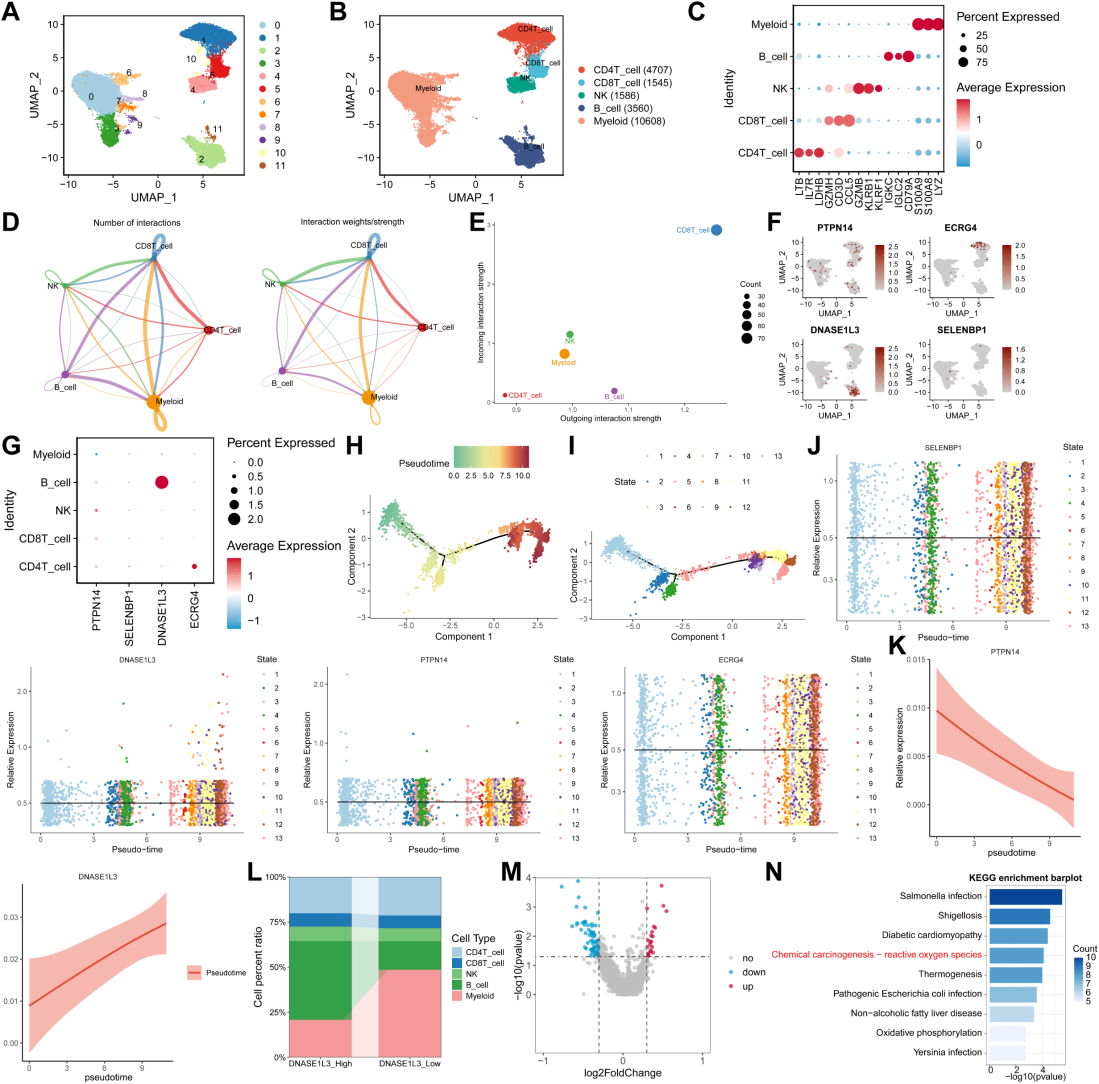
Single-cell analysis reveals expression and functional characteristics of four genes in SLE in the GSE142016 dataset. **(A)** UMAP visualization showing 11 clusters identified. **(B)** UMAP plot annotated with 5 immune cell types. **(C)** Heatmap presenting the top 3 marker genes for each cell type. **(D)** Cell-cell communication network analysis. **(E)** Major signal senders and receivers in cellular communication. **(F)** UMAP plots showing distribution and expression levels of the four genes across 5 cell types. **(G)** Heatmap demonstrating expression patterns of the four genes among 5 cell types. **(H)** Reconstructed pseudotime trajectory. Each point denotes a single cell colored by pseudotime progression. **(I)** Cell state assignment along pseudotime, indicating different branches or differentiation states. **(J)** Developmental trajectories of PTPN14 and DNASE1L3 across pseudotime (ECRG4 and SELENBP1 excluded due to low expression). Line plots show smoothed expression profiles along pseudotime. **(K)** Expression dynamics of PTPN14 and DNASE1L3 along pseudotime. **(L)** Proportional distribution of cell types between DNASE1L3-high and -low expression groups. **(M)** Volcano plot visualizing differentially expressed genes between the two groups. **(N)** KEGG pathway enrichment analysis of DEGs. PTPN14, protein tyrosine phosphatase non-receptor type 14; DNASE1L3, deoxyribonuclease 1 like 3.

Analysis of single-cell RNA sequencing data from 3 SLE patients in the GSE142016 dataset identified 11 clusters, which were annotated into 5 immune cell types, including CD4 T cells, CD8 T cells, NK cells, B cells, and myeloid cells (**Figures 11A, B**). The top three marker genes for each cell type are presented in **Figure 11C**. Cell communication analysis demonstrated that CD8 T cells were both the primary senders and receivers of signals (**Figures 11D, E**, **Supplementary Figure S6**). UMAP plots displayed the distribution and expression levels of the four feature genes across the five cell types (**Figure 11F**). The results showed that DNASE1L3 was mainly expressed in B cells, PTPN14 in NK cells, SELENBP1 in CD4 T cells and myeloid cells, and ECRG4 primarily in CD4 T cells (**Figure 11G**). Pseudotime analysis was conducted (**Figures 11H, I**); however, due to low expression levels of ECRG4 and SELENBP1, only the trajectories of PTPN14 and DNASE1L3 were explored (**Figure 11J**). PTPN14 expression gradually decreased along pseudotime, whereas DNASE1L3 expression progressively increased (**Figure 11K**). Further stratification of all cells into DNASE1L3-high and -low subgroups revealed that B cells were the predominant cell type in the high-expression group, while myeloid cells were most abundant in the low-expression group (**Figure 11L**). A total of 111 DEGs were identified between the two groups (**Figure 11M**). KEGG pathway analysis indicated significant enrichment in salmonella infection, shigellosis, diabetic cardiomyopathy, and chemical carcinogenesis-reactive oxygen species (**Figure 11N**).

### Spatial transcriptomics analysis revealed the expression and localization of four signature genes in CRC

**Figure 12:**
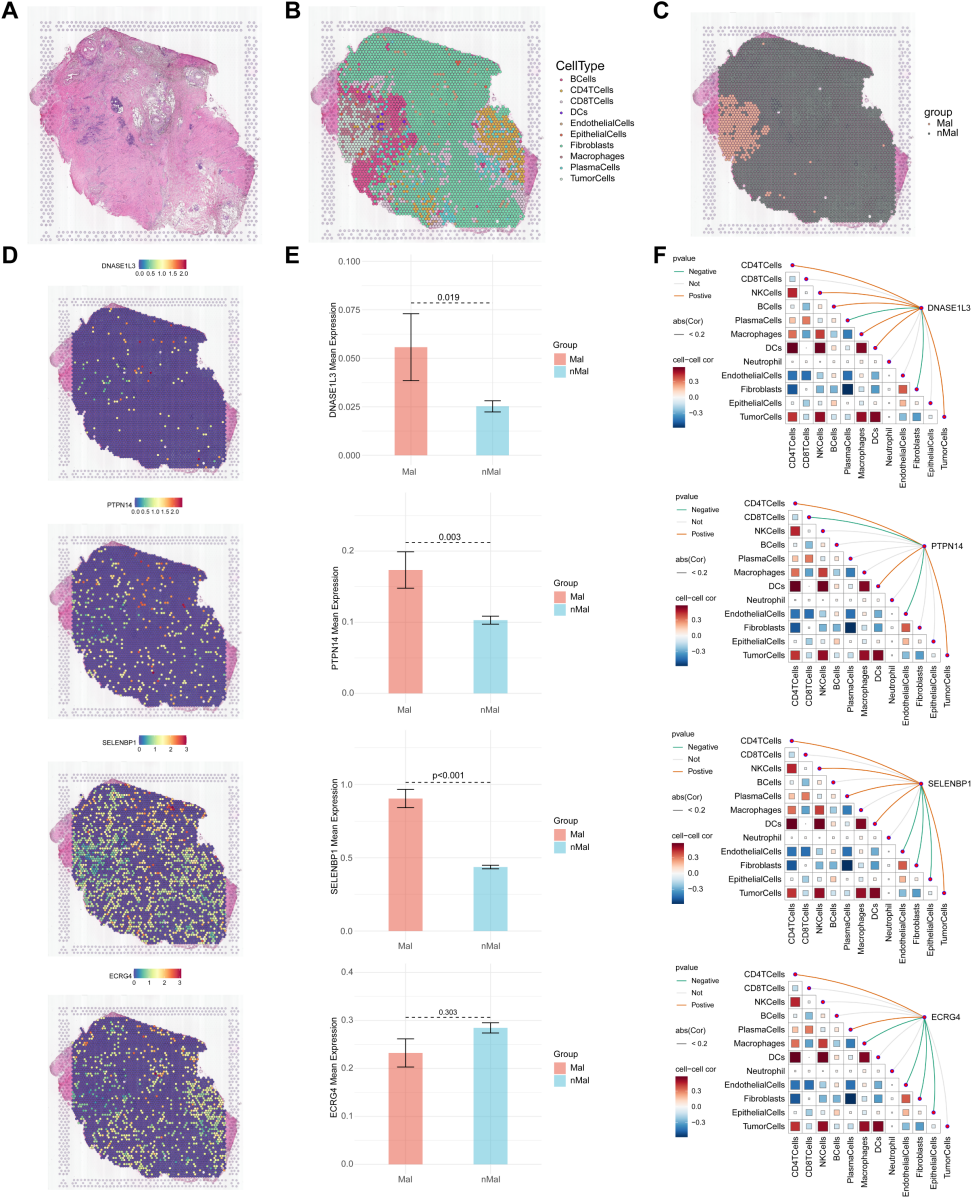
Spatial transcriptome analysis of four genes in CRC pathological tissues. **(A)** H&E staining of pathological sections. **(B)** Spatial localization of ten cell types. **(C)** Distribution of malignant and non-malignant regions. **(D)** Spatial localization of the four genes. **(E)** Comparison of expression levels of the four genes in malignant and non-malignant regions. **(F)** Correlation between expression levels of the four genes and different cell types. The color scale encodes correlation coefficients (r), with red indicating positive and blue indicating negative correlations.

Integrated spatial transcriptome sequencing analysis was performed to explore the spatial location and gene expression of four prognostic genes in CRC tissue. As shown in **Figure 12A**, Hematoxylin and eosin (H&E) staining of pathological sections was performed. Ten distinct cell types were identified, and their spatial localization was presented in **Figure 12B**. The demarcation between malignant and non-malignant regions was displayed in **Figure 12C**. The spatial distribution of the four signature genes was shown in **Figure 12D**. Further investigation into their expression revealed that DNASE1L3, PTPN14, and SELENBP1 were primarily localized to the malignant regions, whereas the expression of ECRG4 showed no significant difference between malignant and non-malignant areas (**Figure 12E**). Correlation analysis demonstrated that all four signature genes were closely associated with a variety of cell types (**Figure 12F**).

### Validation of the expression of prognostic genes for CRC

**Figure 13:**
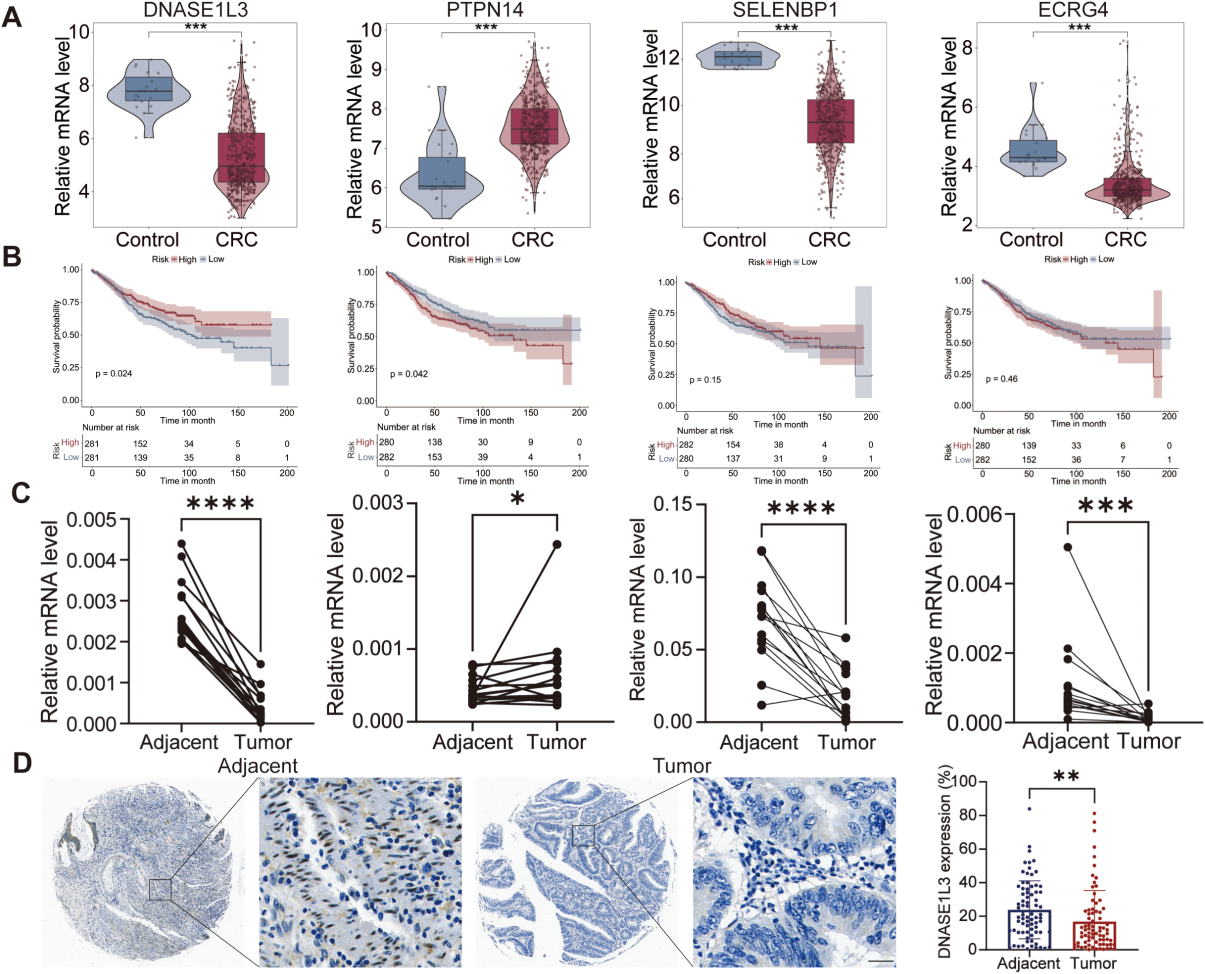
Validation of the expression of four genes in CRC. **(A)** The mRNA expression levels of four hub genes in the GSE39582 dataset. **(B)** Survival comparison between high and low expression groups of four genes in the GSE39582 dataset. **(C)** The mRNA expression levels of four hub genes in the 15 pairs of tumor and adjacent tissues. **(D)** IHC staining of DNASE1L3 protein in tumor tissues and adjacenttissues from 76 CRC patients. Scale bar 25 μm. *p < 0.05; **p < 0.01; ***p < 0.001; ****p < 0.0001.

The mRNA expression levels of DNASE1L3, SELENBP1 and ECRG4 were significantly lower in the tumor group compared to the control group in the GSE39582 dataset, whereas the mRNA expression levels of PTPN14 were significantly higher in the tumor group (**Figure 13A**). Consistent with the aforementioned results, similar expression trends for DNASE1L3, ECRG4, and SELENBP1 were observed in the COAD and READ datasets, except that PTPN14 showed no significant difference in the COAD dataset but was markedly downregulated in the READ dataset (**Supplementary Figure S7**). Patients with low levels of DNASE1L3 exhibited a significantly poorer prognosis than those with high levels, whereas the opposite trend was observed for PTPN14. In contrast, the expression of SELENBP1 and ECRG4 showed no significant association with patient survival (**Figure 13B**). The qPCR results of the 15 pairs of tumor and pericarcinomatous tissues confirmed the above results (**Figure 13C**).

These findings suggest that DNASE1L3 and PTPN14 may represent potential key targets for improving prognosis in CRC. Given that immune dysregulation is a critical pathological feature shared by CRC and SLE, our study, along with existing evidence, indicates that DNASE1L3—primarily expressed in macrophages—plays a central role in mediating the clearance of apoptotic cells, maintaining self-antigen homeostasis, and preventing immune dysregulation. Additionally, IHC staining of tumor tissues and pericarcinomatous tissues from 76 CRC patients further confirmed the lower expression level of DNASE1L3 protein in tumor tissues (**Figure 13D**).

### Validation of the expression of diagnostic genes for SLE

**Figure 14:**
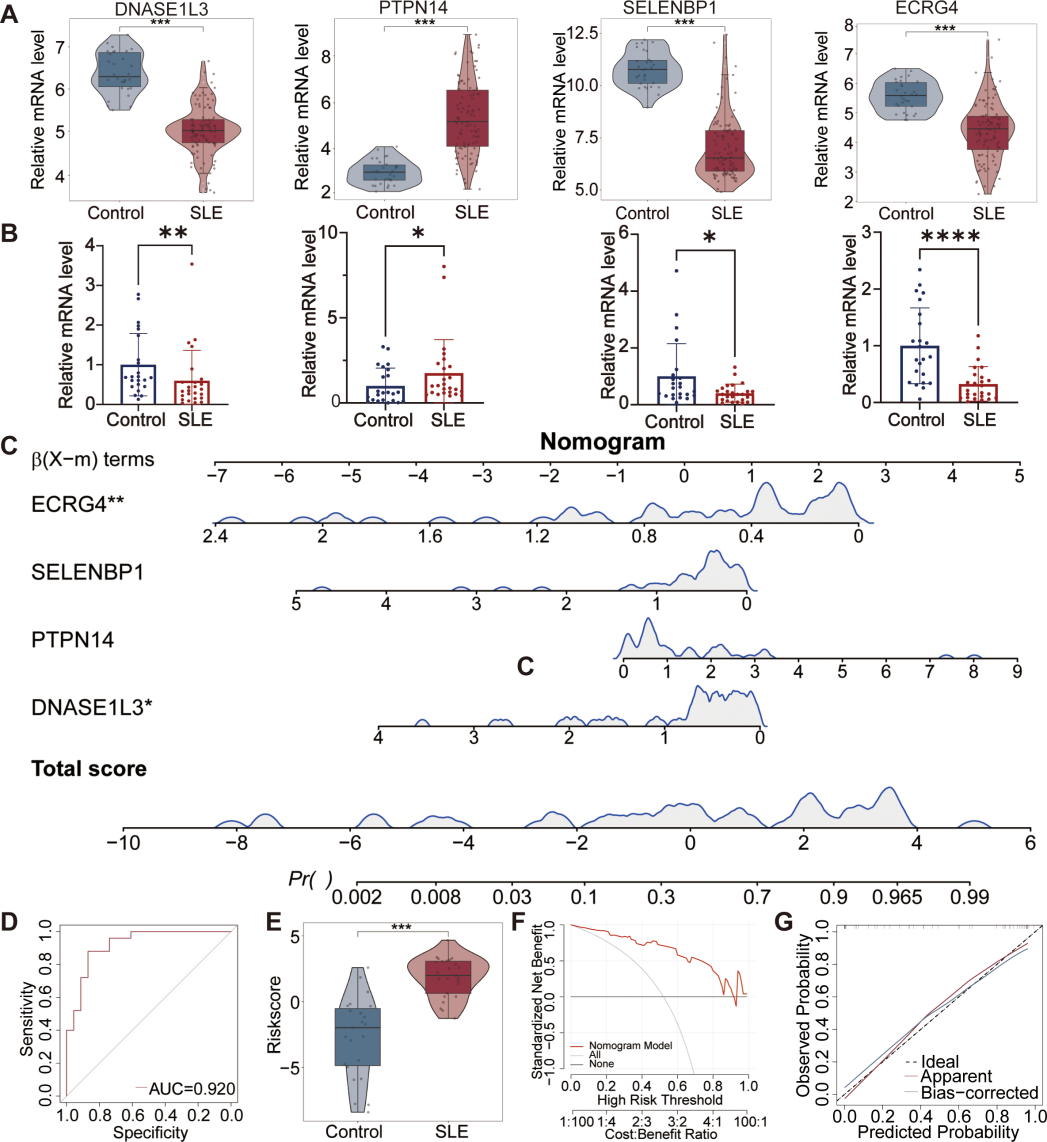
Validation of the expression of four genes in SLE. **(A)** The mRNA expression levels of four genes in the GSE61635 dataset. **(B)** The mRNA expression levels of four genes of PBMCs from 25 SLE patients and 23 controls. **(C)** Nomogram model for diagnosing SLE based on expression levels of four genes. **(D)** ROC curve showing an AUC of 0.920 for the model in diagnosing SLE. **(E)** Violin plot demonstrating significantly higher risk scores in SLE compared to controls. **(F)** DCA curve of the model. **(G)** Calibration curve indicating close alignment between predicted and observed probabilities of the model. *p < 0.05; **p < 0.01; ***p < 0.001; ****p < 0.0001. PBMC, peripheral blood mononuclear cell.

The expression levels of DNASE1L3, PTPN14, SELENBP1, and ECRG4 in the GSE61635 dataset were consistent with those in the GSE39582 dataset (**Figure 14A**). Similarly, RT-qPCR analysis of PBMCs from 25 SLE patients and 23 healthy controls confirmed the mRNA expression levels of these four genes (**Figure 14B**). Subsequently, a diagnostic nomogram was developed to predict the probability of SLE development in both the control and SLE group (**Figure 14C**). The ROC curve demonstrated that the nomogram achieved an AUC of 0.920 for diagnosing SLE (**Figure 14D**). Nomogram assessment revealed a significant discrimination in SLE risk between the control and SLE groups (**Figure 14E**). Both the calibration and DCA curves indicated that nomogram-based decisions could provide clinical utility for SLE prediction (**Figures 14F, G**).

### Low levels of DNASE1L3 impair macrophage phagocytic function.

**Figure 15:**
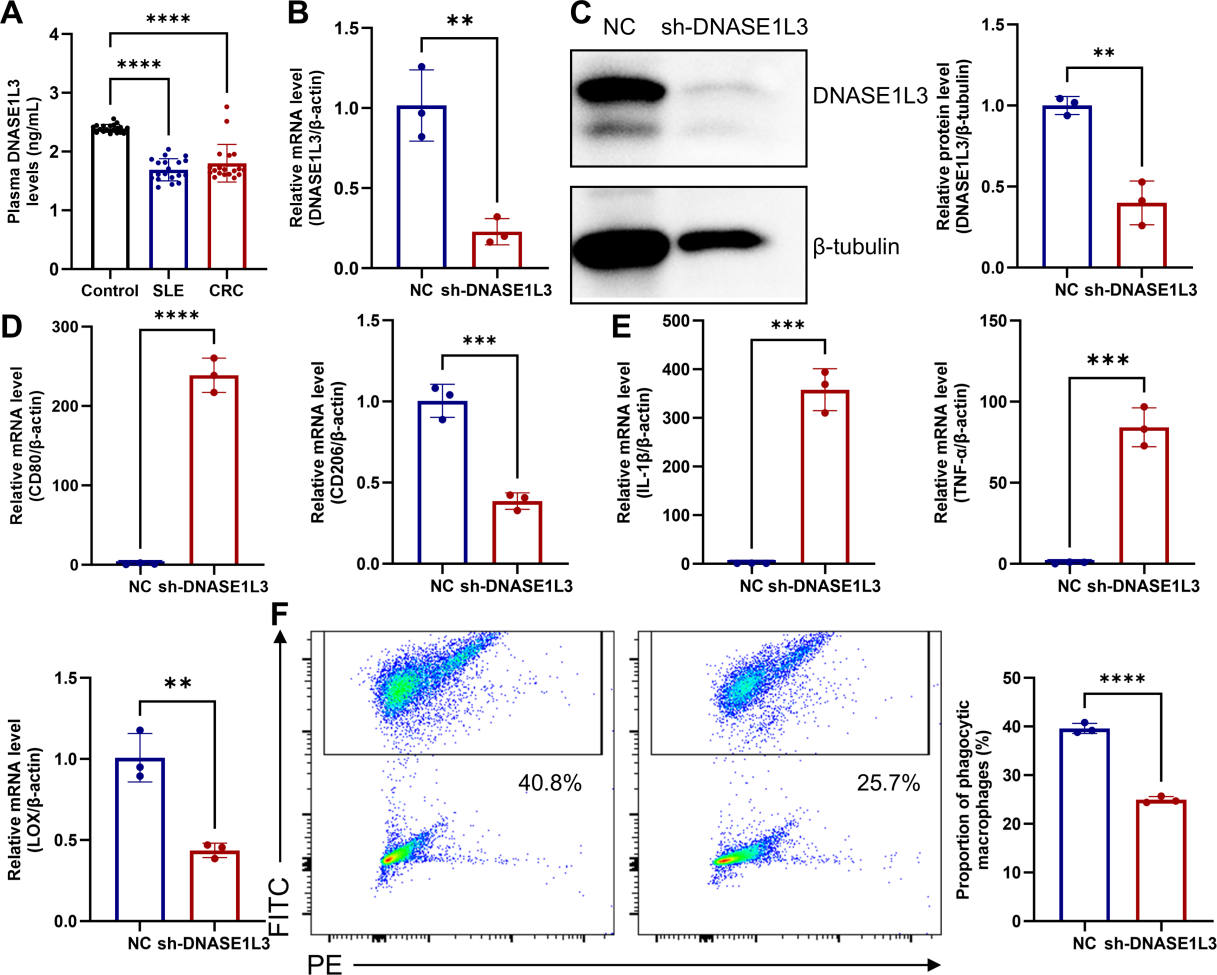
Low levels of DNASE1L3 impair macrophage phagocytic function. **(A)** ELISA analysis displaying reduced DNASE1L3 levels in SLE and CRC groups compared to the control group. The efficiency of DNASE1L3 knockdown in THP-1 cells was validated by RT-qPCR **(B)** and Western blotting **(C)** analyses. RT-qPCR results indicated that knockdown of DNASE1L3 influenced THP-1 cell differentiation **(D)** and the expression of cytokines and enzymes **(E)**. **(F)** Flow cytometry analysis demonstrated the effect of DNASE1L3 knockdown on efferocytosis in THP-1 cells. **p < 0.01; ***p < 0.001; ****p < 0.0001.

Significantly reduced DNASE1L3 levels were observed in both the SLE and CRC groups compared to the control group via ELISA method (**Figure 15A**). To simulate the pathological microenvironment of low DNASE1L3 expression observed in both diseases, we knocked down DNASE1L3 expression in the human monocytic cell line THP-1 to further investigate the regulatory mechanisms of this gene in disease pathogenesis. RT-qPCR and Western blotting were used to verify the knockdown efficiency of DNASE1L3 in the constructed THP-1 cell line (**Figures 15B, C**). RT-qPCR results demonstrated that low levels of DNASE1L3 promoted the differentiation of THP-1 cells toward the M1 phenotype while suppressing their differentiation into M2 macrophages (**Figure 15D**). Concurrently, there was a significant increase in IL-1β and TNF-α secreted by M1 macrophages, along with a marked reduction in LOX protein expression by M2 macrophages (**Figure 15E**). Flow cytometric analysis confirmed that reduced DNASE1L3 impaired macrophage efferocytosis (**Figure 15F**).

## Discussion

Through its roles of defense, immune surveillance, and maintenance of homeostasis, the immune system maintains the stability of the internal environment. Immune dysregulation can lead to various diseases, such as cancer, autoimmune diseases, and infections (39). Autoimmune diseases are characterized by a loss of self-tolerance and disruption of immune homeostasis (40). Conversely, insufficient immune responses can contribute to tumor development, which is often accompanied by inflammatory reactions. SLE and CRC are common immune-related diseases whose pathogenic mechanisms have not been fully elucidated. Although they appear to share potential common immune mechanisms, their immunological characteristics remain to be systematically summarized. Therefore, we constructed a shared differential gene expression profile of SLE and CRC to explore the commonalities between autoimmune diseases and cancer.

Through integrated multi-omics analysis, this study reveals that SLE and CRC share two core pathological features, characterized by impaired immune clearance and disrupted metabolic homeostasis. Key evidence indicates that *DNASE1L3*, *PTPN14*, *SELENBP1*, and *ECRG4* exhibit consistent dysregulation in both diseases, and a model constructed based on these genes demonstrates robust performance in both SLE and CRC cohorts. This discovery provides a novel perspective for understanding the shared mechanisms between autoimmune diseases and cancer.

We initially identified 58 common DEGs between SLE and CRC. These genes were significantly enriched in biological processes such as apoptosis, cell cycle regulation, immune cell activation, and metabolic reprogramming. Drug sensitivity prediction analysis suggested that targeting histone deacetylases and the PI3K/AKT signaling pathway may help modulate the aberrant gene expression patterns in both diseases. Notably, immune infiltration analysis revealed that SLE and CRC shared decreased proportions of resting memory CD4^+^ T cells and resting mast cells, along with increased infiltration of neutrophils and M0 macrophages. Furthermore, the expression levels of the four genes were closely associated with infiltrating levels of various immune cells, including T cells, macrophages, neutrophils, and mast cells. This shared immune microenvironment composition provides a cellular basis for the co-morbidity development between these two diseases.

By integrating single-cell RNA sequencing and spatial transcriptomics, we delineated the cell type-specific distribution of the four genes. Spatial transcriptomic analysis revealed that PTPN14 and SELENBP1 were highly expressed in malignant regions, whereas ECRG4 was predominantly localized to non-malignant areas. Single-cell resolution further demonstrated that PTPN14 was mainly distributed in epithelial cells in CRC and in NK cells in SLE. SELENBP1 was primarily expressed in epithelial cells and fibroblasts in CRC, and in myeloid cells and CD4^+^ T cells in SLE. ECRG4 was largely detected in fibroblasts in CRC and in CD4^+^ T cells in SLE. PTPN14, a non-receptor type protein tyrosine phosphatase, participates in the regulation of cell migration, differentiation, and adhesion by mediating dephosphorylation of proteins such as YAP (41). Furthermore, studies have shown that PTPN14 promotes tumor progression via the PI3K/AKT/mTOR pathway (42). SELENBP1 modulates protein transport and degradation and has been documented to be dysregulated in in several autoimmune diseases. Moreover, it exerts tumor-suppressive effects by inhibiting the activity of hypoxia-inducible factor-1 alpha (43). ECRG4, also known as C2orf40, is primarily localized to the cell membrane. In CRC cells, ECRG4 is highly methylated, leading to transcriptional silencing. Its expression promotes apoptosis and inhibits proliferation (44). Additionally, ECRG4 is expressed in polymorphonuclear cells and helps maintain cellular homeostasis. At sites of inflammation or tumor growth, it is cleaved by proteases such as thrombin, releasing bioactive peptides with pro-inflammatory activity that recruit neutrophils to the site of injury and contribute to tissue repair (45).

This study revealed a consistent reduction in both intracellular and extracellular DNASE1L3 protein levels in patients with SLE and CRC, implying that this downregulation could be involved in the immune dysregulation common to both conditions. DNASE1L3 is an extracellular nuclease primarily highly expressed in in macrophages and dendritic cells, involved in clearance of extracellular and apoptotic DNA, suggesting its potential relevance in autoimmune diseases and cancer. In SLE, impaired clearance of apoptotic cells leads to the accumulation of phospholipids and nucleic acids, which act as autoantigens that activate B cells and promote the production of autoantibodies such as anti-phosphatidylserine and anti-dsDNA (46). Concurrently, the phagocytic clearance of immune complexes by phagocytes is diminished, resulting in the deposition of circulating immune complexes in target organs and subsequent tissue damage (47). In CRC, genomic instability and immune evasion are key pathogenic features. Dysfunctional DNASE1L3 may contribute to the accumulation of DNA fragments, chronic inflammation, and disruption of immune tolerance, suggesting a potential shared pathological mechanism in both diseases (48). Our experimental data further demonstrated that DNASE1L3 deficiency impairs the ability of phagocytes to clear apoptotic cells, thereby jointly promoting the pathogenesis of SLE and CRC.

Macrophages reside in nearly all tissues and play diverse roles in processes such as microbial defense, tissue repair, inflammation, and metabolic disorders. They exhibit remarkable functional plasticity and serve as a critical bridge between innate and adaptive immunity (49). Dysfunctional macrophages represent a common pathological feature of both SLE and CRC. Numerous studies have confirmed that in SLE, macrophages exhibit upregulation of autophagy- and apoptosis-related genes, while deficiency in peroxisome proliferator-activated receptor gamma (PPARγ) prevents their acquisition of an anti-inflammatory phenotype. This leads to impaired phagocytic capacity and exacerbates target organ damage (50). Building on these findings, our study further revealed that low DNASE1L3 expression disrupts the M1/M2 polarization balance in macrophages, impairs their ability to clear apoptotic cells, and may contribute to the accumulation of autoantigens, thereby triggering inflammatory responses and immune dysregulation.

We identified DNASE1L3 as a key protective factor involved in both SLE and CRC. Due to immune dysregulation, SLE can progress to involve neuropsychiatric symptoms, nephritis, enteritis, and dermatitis. Previous studies have confirmed shared microbial features and functional alterations between SLE and inflammatory bowel disease (51). Functional deficiency of DNASE1L3 impairs systemic and intestinal tolerance to extracellular DNA, allowing apoptotic fragments to amplify immune responses and promote inflammation. Multiple studies have reported DNASE1L3 as a susceptibility gene for SLE, with deficient patients exhibiting elevated plasma DNA levels (52). Elevated anti-DNASE1L3 antibodies are closely associated with SLE, and DNASE1L3-deficient mice develop lupus-like phenotypes (53). Several studies have reported downregulation of DNASE1L3 in CRC cells, promoting proliferation and migration. In an azoxymethane/dextran sulfate sodium (AOM/DSS) model of inflammation-induced colorectal cancer, DNASE1L3-knockout mice exhibited more severe colonic epithelial damage, increased hemorrhage, greater weight loss, diarrhea, and larger tumor volumes compared to wild-type mice (54). These findings further support that DNASE1L3 deficiency promotes tumor initiation and exacerbates progression (54). This study constructed a four-gene-based model for the early diagnosis of SLE and CRC, and also providing prognostic evidence for CRC. Furthermore, we proposed a cross-disease model centered on DNASE1L3 dysregulation to explain the shared mechanisms between autoimmune diseases and cancer.

This study has several limitations. Due to batch differences among various public datasets and cohort-specific confounding factors, the observed gene modules and enriched pathways may be significantly affected. Our integrative analyses cannot robustly stratify patients by disease stage, specific clinical phenotypes, molecular subtypes, or treatment exposure. Moreover, the clinical samples used for ELISA and RT-qPCR were limited. The current work does not determine whether the observed patterns are broadly generalizable or driven by distinct subgroups. We used PMA-differentiated THP-1 cells, a human monocytic leukemia line, as an *in vitro* macrophage model to examine the impact of DNASE1L3 deficiency on efferocytosis and polarization. While THP-1 cells are widely employed for mechanistic studies, their malignant origin is an inherent limitation. Our current results should therefore be viewed as proof-of-concept. Future experiments using RAW264.7 cells and primary macrophages derived from human PBMCs are underway to validate the effects of DNASE1L3 on macrophage polarization and efferocytic capacity in more physiologically relevant systems. While the *in vitro* THP-1 model aligns with the inflammatory profiles of SLE and CRC, direct *in vivo* validation (e.g., using specific knockout mouse models for both diseases) remains a necessary future step to confirm this axis operates physiologically to drive disease progression. In the future, we will conduct large-sample prospective studies to evaluate the clinical utility of the four-gene model. At the same time, we will carry out a series of basic research projects to systematically investigate the role of DNASE1L3-related pathways in SLE and CRC.

## Conclusion

Through an integrated multi−omics approach, this study identified a four−gene model based on DNASE1L3, PTPN14, SELENBP1, and ECRG4, which may have potential utility for early identification and risk stratification in both SLE and CRC. In addition, DNASE1L3−related pathways may represent an interesting focus for future mechanistic and translational research in both diseases.

## Data Availability

The data presented in the study are deposited in the Zenodo repository, accession number 10.5281/zenodo.18753102.
