# Supplementary material for: Shared immune dysregulation in systemic lupus erythematosus and colorectal cancer: a multi-omics guided discovery of DNASE1L3-centric efferocytosis deficiency
Source: Front Immunol. 2026 Feb 26;17:1775776. doi: 10.3389/fimmu.2026.1775776 (PMC12979178; doi:10.3389/fimmu.2026.1775776)
Supplement: Supplementary file 1 [file DataSheet1.docx]

**Supplemental materials**

Inventory of supplementary materials

1. Table S1………….…………………Page 2
2. Figure S1………….………………. Page 3
3. Figure S2………….………………. Page 4
4. Figure S3………….………………. Page 5
5. Figure S4………….………………. Page 6
6. Figure S5………….………………. Page 7
7. Figure S6………….………………. Page 8
8. Figure S7………….………………. Page 9

## Table S1 Sequences of the primers used for PCR.

| Gene | Species |  | Sequence |
| --- | --- | --- | --- |
| DNASE1L3 | Homo | Forward | TGGTTGAGGTCTACACGGACGT |
|  |  | Reverse | GTCAGTCCTCAAGCGGATGTTC |
| PTPN14 | Homo | Forward | AGTGTGGTGAGCACTACTCGGA |
|  |  | Reverse | CTACACACGCTGCCATTGGTGA |
| SELENBP1 | Homo | Forward | TTGGAGATCCGCTTCCTGCACA |
|  |  | Reverse | GGATCACCTTCTCCACTGACCA |
| ECRG4 | Homo | Forward | CCAGCAGTTTCTCTACATGGGC |
|  |  | Reverse | GCAGAGTCTTCATCATAGTGACG |
| CD80 | Homo | Forward | CTCTTGGTGCTGGCTGGTCTTT |
|  |  | Reverse | GCCAGTAGATGCGAGTTTGTGC |
| CD206 | Homo | Forward | AGCCAACACCAGCTCCTCAAGA |
|  |  | Reverse | CAAAACGCTCGCGCATTGTCCA |
| IL-1β | Homo | Forward | TTCGAGGCACAAGGCACAA |
|  |  | Reverse | TGGCTGCTTCAGACACTTGAG |
| TNF-α | Homo | Forward | GCTGCACTTTGGAGTGATCG |
|  |  | Reverse | GCTTGAGGGTTTGCTACAACA |
| LOX | Homo | Forward | GATACGGCACTGGCTACTTCCA |
|  |  | Reverse | GCCAGACAGTTTTCCTCCGCC |
| β-actin | Homo | Forward | GAAGAGCTACGAGCTGCCTGA |
|  |  | Reverse | CAGACAGCACTGTGTTGGCG |


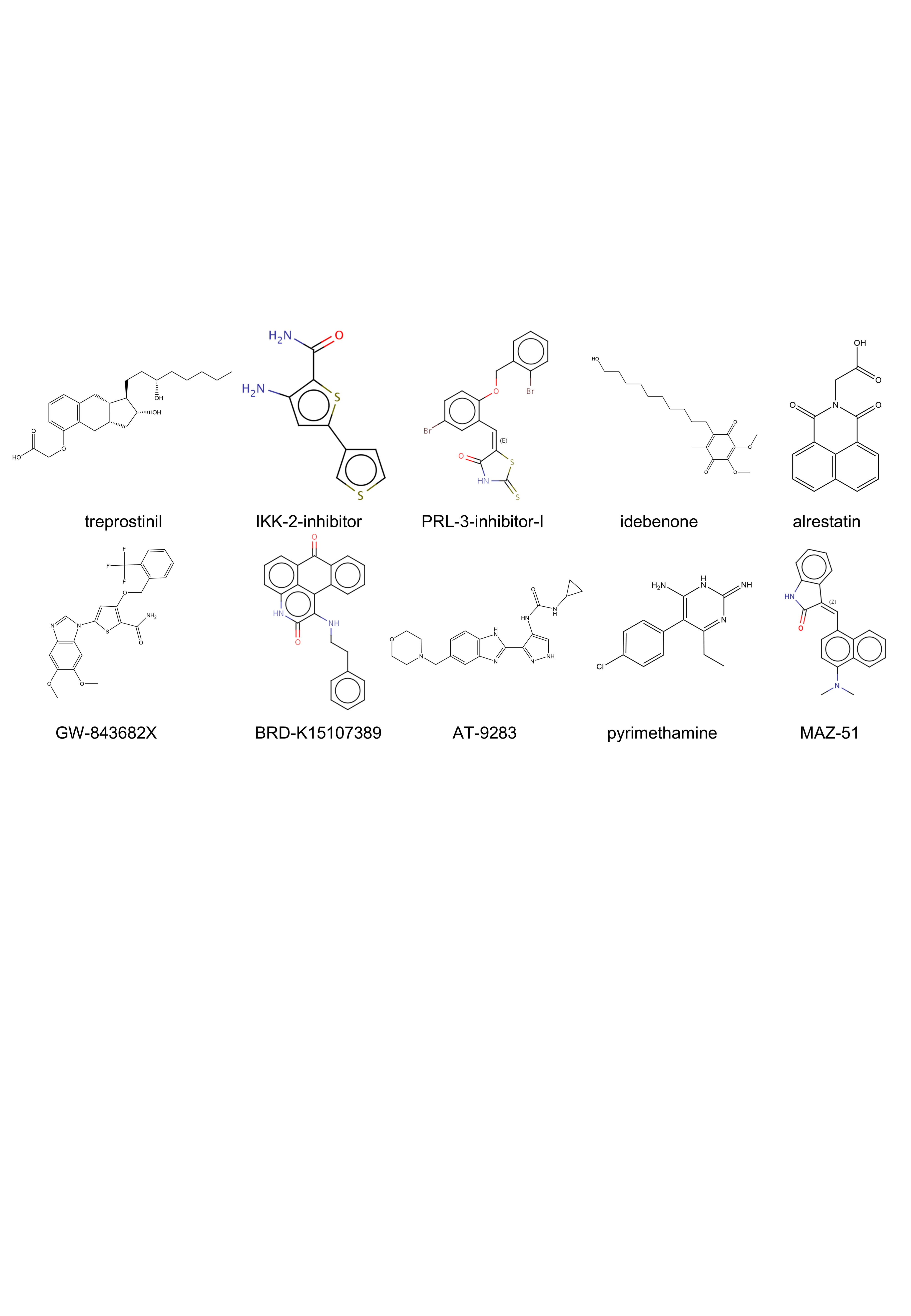


**Figure S1** The chemical structures of top10 compounds for CRC and SLE treatment.


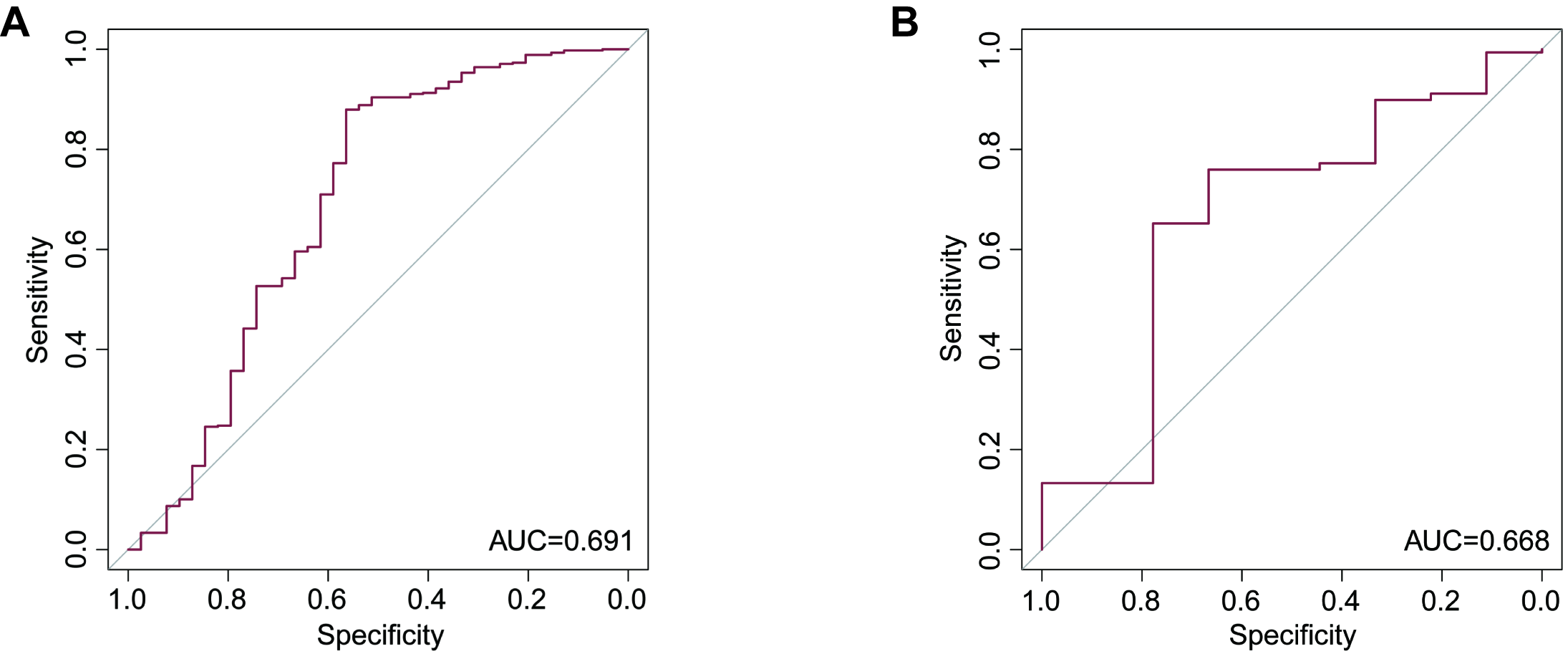


**Figure S2** ROC curves of the 4-gene-based risk score for diagnosing CRC. ROC curve of the risk score for diagnosing CRC in the COAD (A) and READ cohort (B).


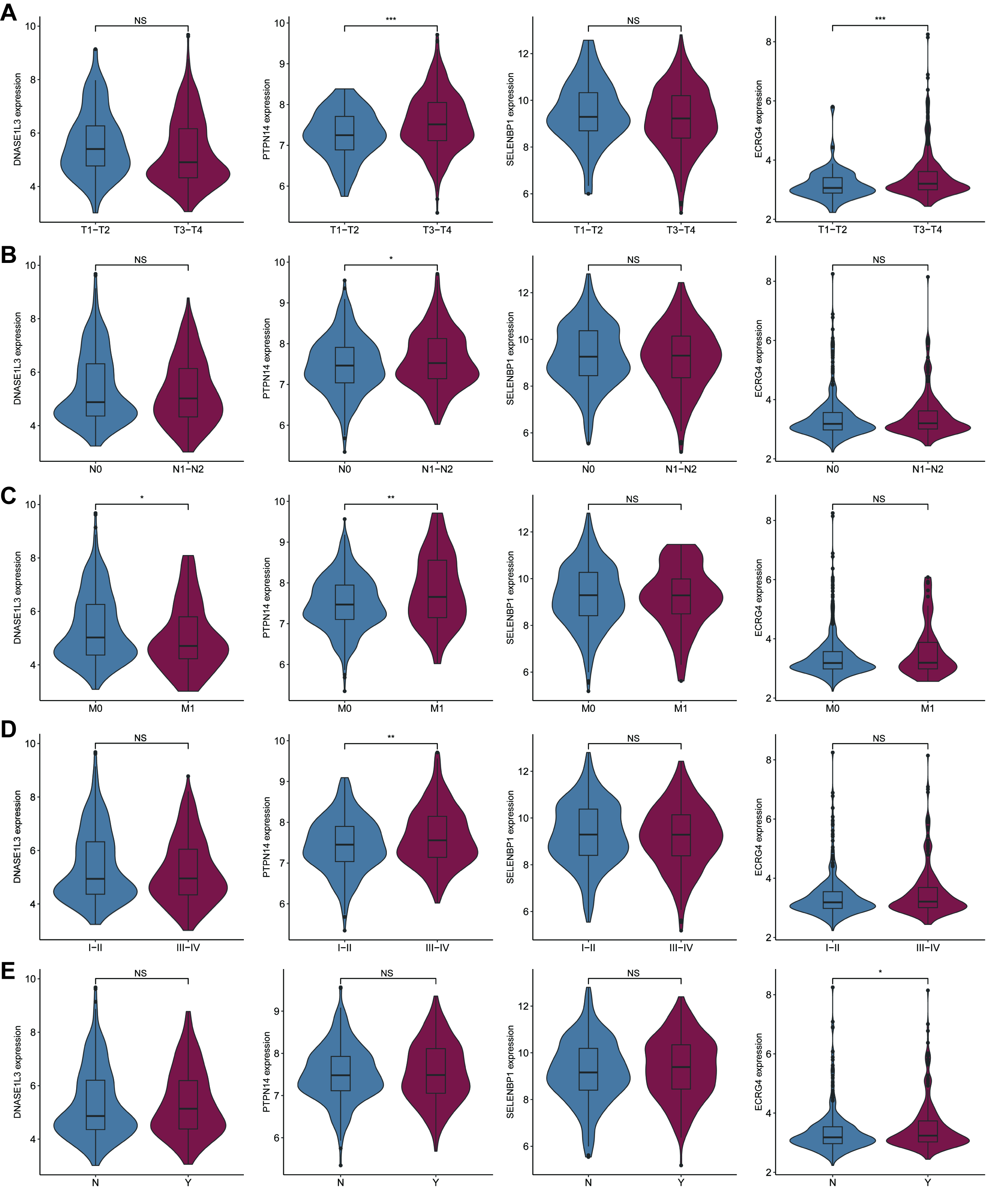


**Figure S3** Expression levels of the four genes in different clinical subgroups. Comparison of the expression levels of the four genes (DNASE1L3, PTPN14, SELENBP1, and ECRG4) according to (A) T classification, (B) N classification, (C) M classification, (D) clinical stage, and (E) chemotherapy status. * p < 0.05; ** p < 0.01; *** p < 0.001; ns not significant.


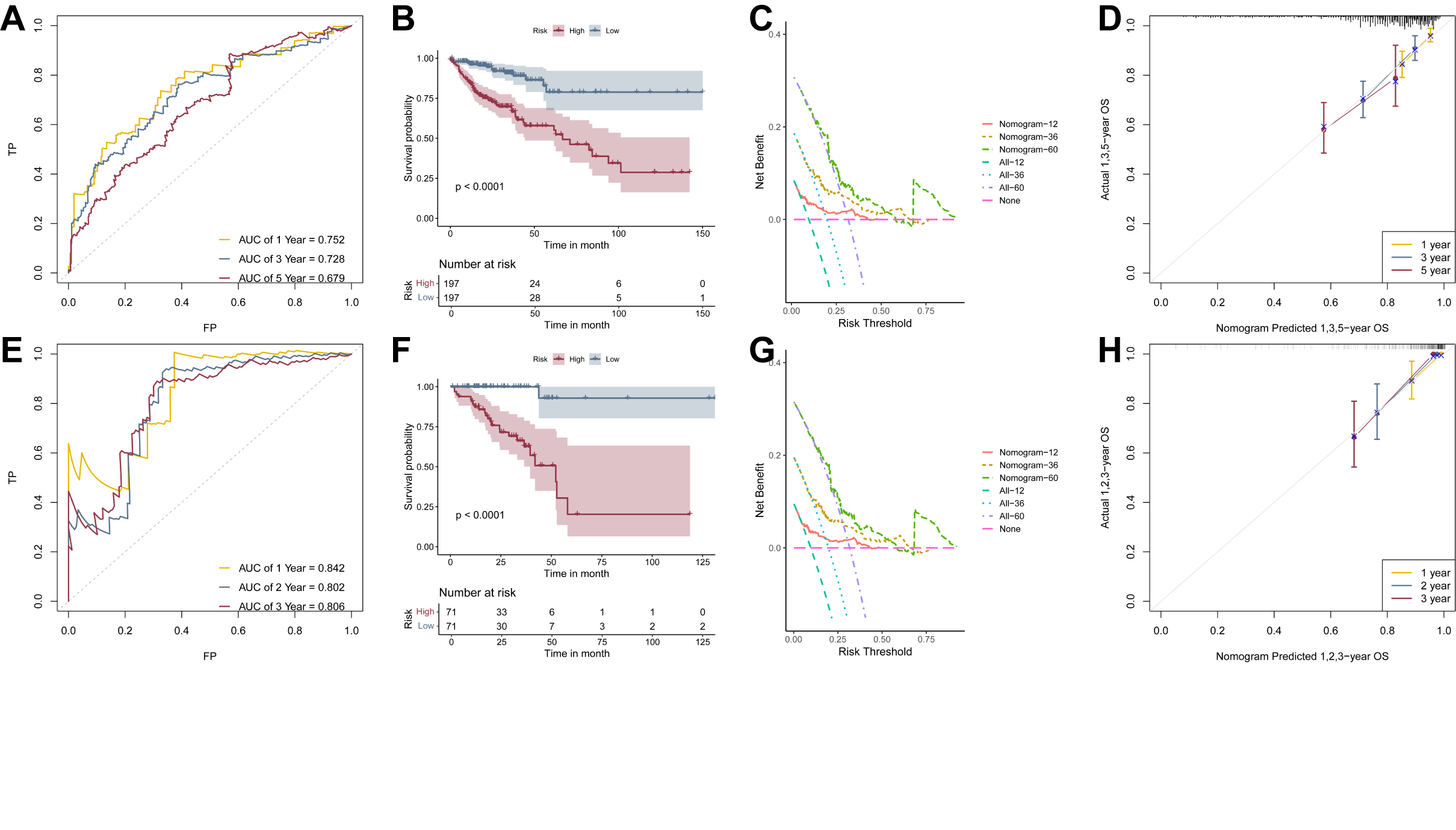


**Figure S4** Validation of prognostic nomogram model for COAD and READ. ROC (A), Kaplan-Meier survival (B), DCA (C), and calibration (D) curves assessing the performance of the nomogram model in predicting OS in the COAD cohort. ROC (E), Kaplan-Meier survival (F), DCA (G), and calibration (H) curves assessing the performance of the nomogram model in predicting OS in the READ cohort.


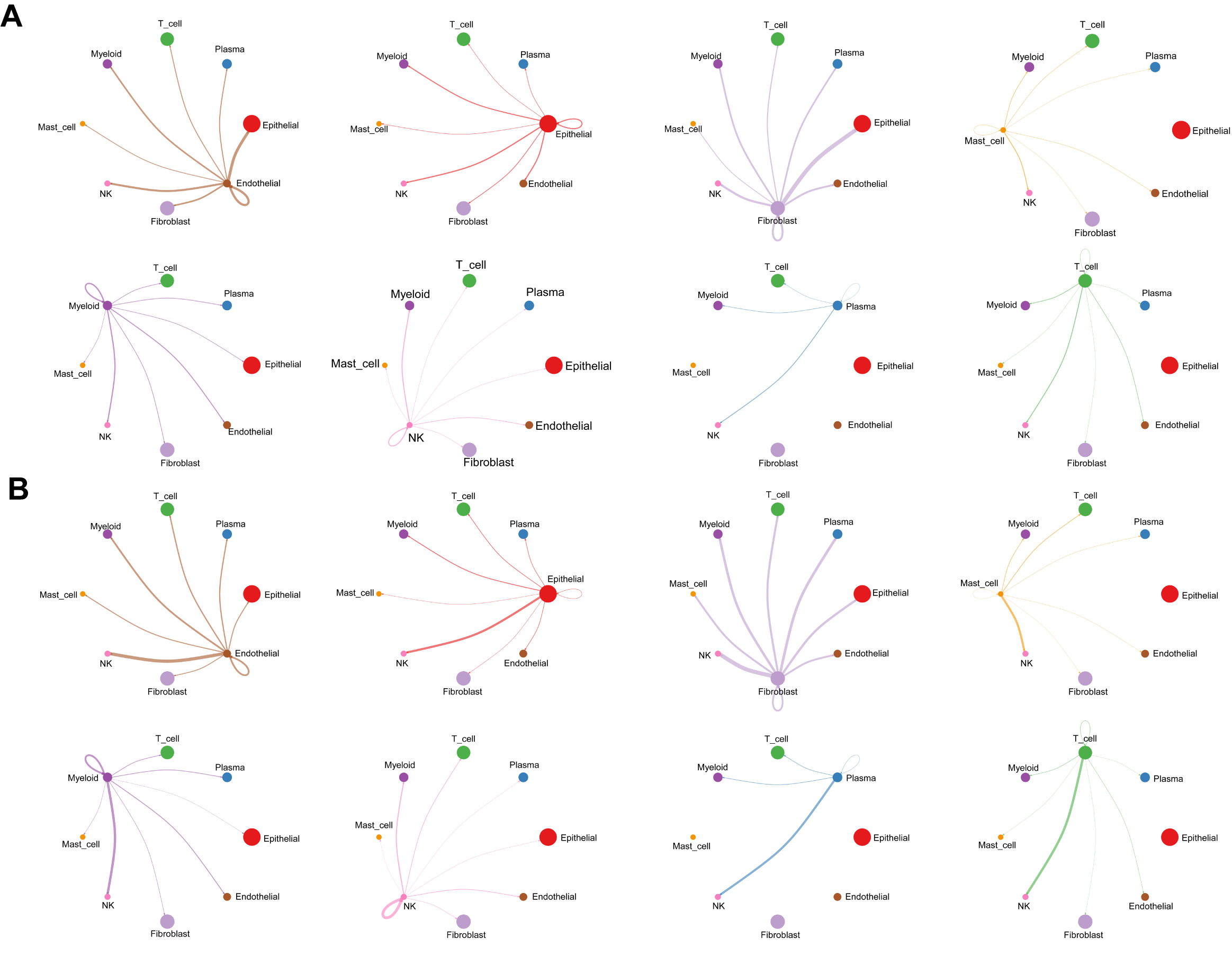


**Figure S5** Single-cell analysis of CRC. Count (A) and weight (B) analysis of intercellular communication networks for each cell type.


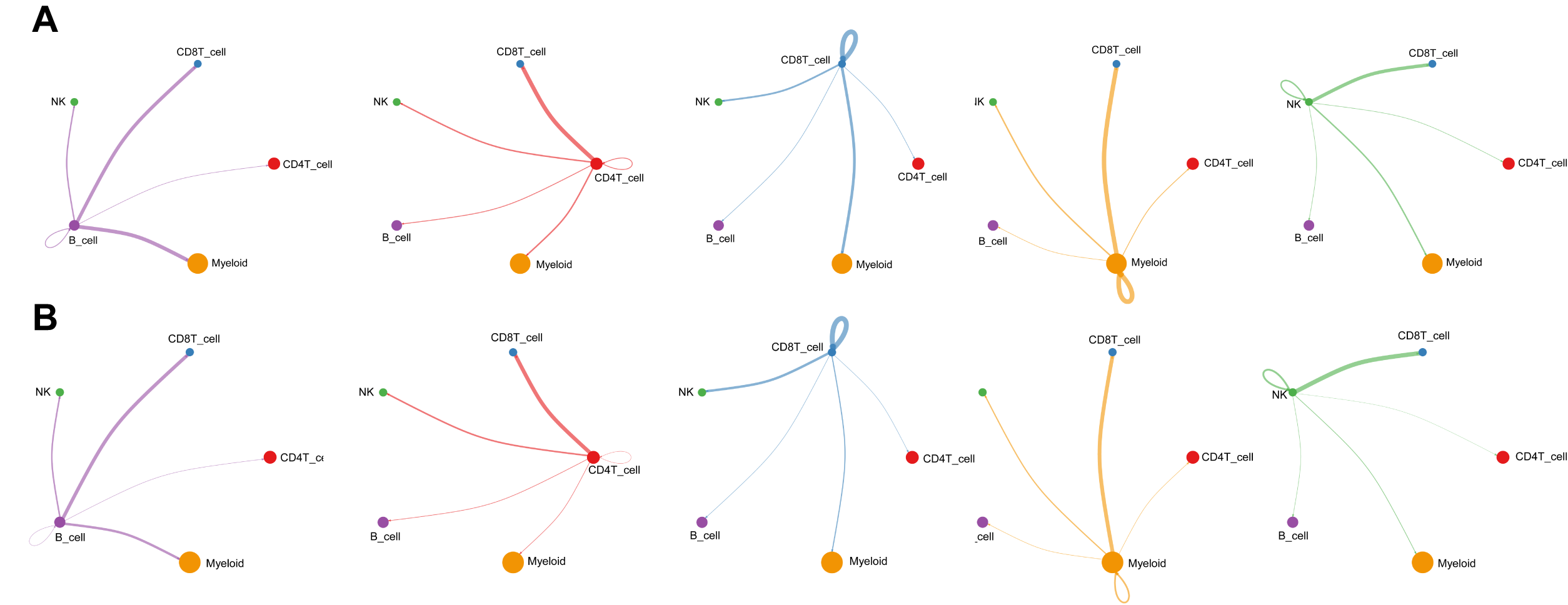


**Figure S6** Single-cell analysis of SLE. Count (A) and weight (B) analysis of intercellular communication networks for each cell type.


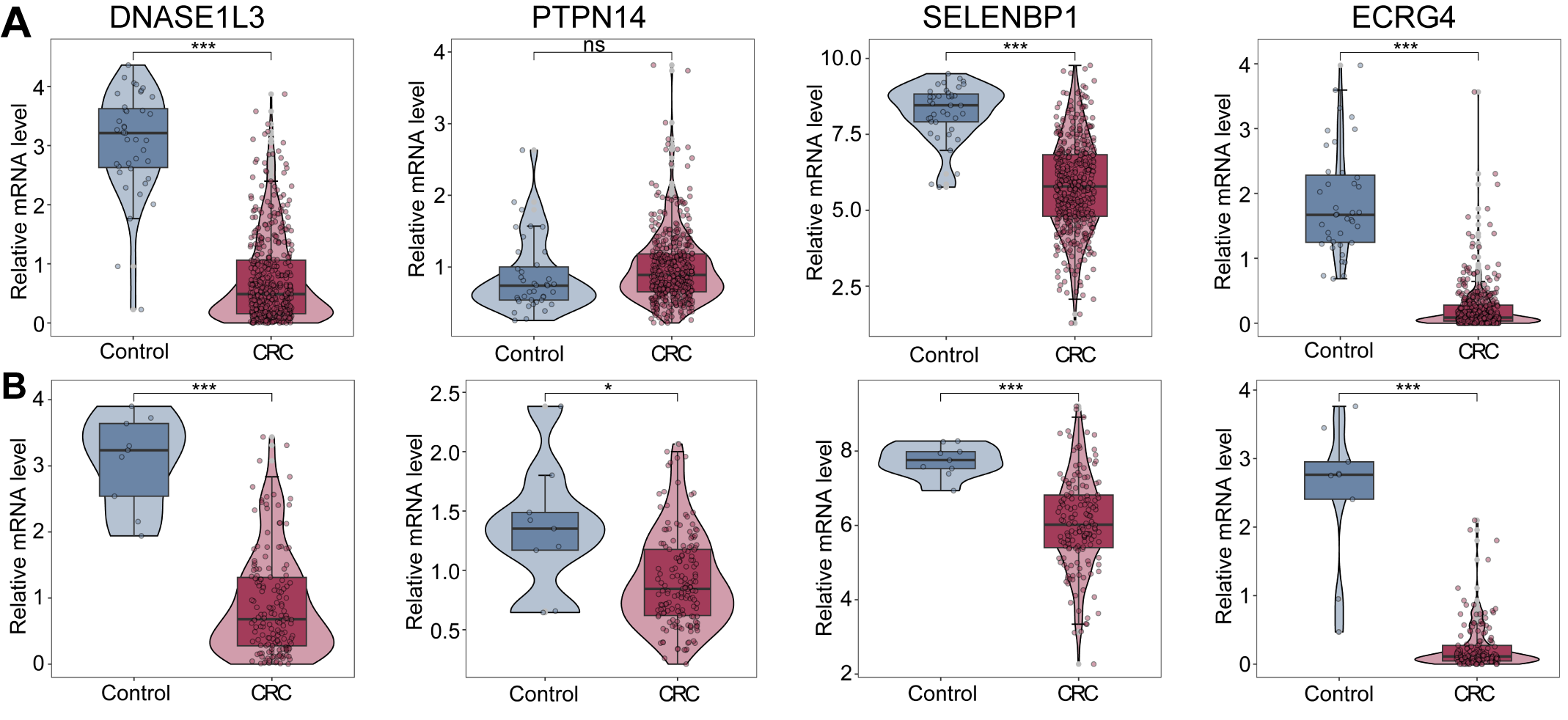


**Figure S7** Expression levels of four genes in COAD and READ datasets. The mRNA expression levels of four hub genes in the COAD (A) and READ datasets.
